# Diabetes impairs antifungal immunity and drives fatal disseminated sporotrichosis in mice

**DOI:** 10.3389/fimmu.2026.1902740

**Published:** 2026-09-03

**Authors:** Mariana Araujo Oliveira, Ywa Perpétuo Socorro Toda Tavares, Renata Chaves Albuquerque, Silene Migliorini, Sandro Rogério Almeida, Joilson O. Martins

**Affiliations:** 1 Laboratory of Immunoendocrinology, Department of Clinical and Toxicological Analyses, School of Pharmaceutical Sciences, University of São Paulo, São Paulo, Brazil; 2 Laboratory of Mycology, Department of Clinical and Toxicological Analyses, School of Pharmaceutical Sciences, University of São Paulo, São Paulo, Brazil

**Keywords:** antifungal immunity, autophagy, diabetes, hyperglycaemia, Sporothrix brasiliensis, sporotrichosis

## Abstract

**Introduction:**

This study establishes and characterizes a novel experimental mouse model to evaluate *Sporothrix brasiliensis* infection under type 1 diabetic conditions, providing a comprehensive description of the associated clinicopathological and immunometabolic alterations.

**Methods:**

Diabetic and normoglycemic control mice were infected with *S. brasiliensis* to evaluate clinical progression, fungal dissemination, mortality, systemic cytokines, splenic T cell subsets, macrophage functional responses, and hepatic autophagic markers (*p62/SQSTM1*, Beclin-1, and LC3B-II).

**Results:**

Diabetic mice exhibited enhanced susceptibility to infection compared to normoglycemic controls, presenting with progressive ulcerative lesions, systemic dissemination (lymph nodes, liver, and brain), and 100% mortality. This severe phenotype was accompanied by a dysregulated inflammatory profile, characterized by elevated systemic TNF-α, IFN-γ, and IL-6 alongside reduced splenic CD4+ T cell frequencies. Functionally, macrophages from diabetic hosts displayed an initial increase in yeast association followed by a rapid decline, consistent with cellular exhaustion. Additionally, infected diabetic mice exhibited hepatic accumulation of *p62/SQSTM1* without alterations in Beclin-1 or LC3B-II, suggesting potential disruption during late-stage autophagic clearance.

**Discussion:**

Overall, this model successfully reproduces severe, disseminated sporotrichosis under hyperglycemic conditions and delineates the multifaceted immune dysregulation associated with host susceptibility. This novel experimental platform provides a valuable tool for future studies exploring fungal pathogenesis and therapeutic strategies in metabolic comorbidities.

## 1 Introduction

Diabetes *mellitus* (DM) is a growing global health concern, with prevalence expected to rise substantially over the coming decades, reaching an estimated 852.5 million people by 2050 (1). Beyond its well-established metabolic and vascular complications, diabetes is increasingly recognized as a condition associated with heightened susceptibility to infection (1, 4–6). Chronic hyperglycaemia disrupts immunometabolic homeostasis by promoting oxidative stress, persistent inflammatory signaling and cellular dysfunction, which together impair both innate and adaptive immunity (2, 3). Concomitantly, diabetes-related microvascular abnormalities may limit immune cell delivery to infected tissues, further weakening local antimicrobial defense (4–6).

Sporotrichosis is an emerging fungal disease caused by dimorphic fungi of the genus *Sporothrix* (7, 8). A defining hallmark of *Sporothrix* biology is its thermal dimorphism: it exists in a saprophytic filamentous phase in the environment and *in vitro* at 25 °C, but transitions into a pathogenic yeast form when exposed to host temperatures (37 °C) *in vivo* or *in vitro* (7, 9). In Brazil, *Sporothrix brasiliensis* is the most prevalent and virulent species within the clinical clade, driven primarily by feline zoonotic transmission (7, 8, 10). Infected cats inoculate high fungal loads via scratches or bites, deeply compromising host subcutaneous tissue (11). The high virulence of *S. brasiliensis* is intrinsically linked to its yeast phase, whose marked adaptability to subcutaneous tissue impairs host immune clearance and facilitates infection establishment (10). Cutaneous manifestations remain the most common clinical presentation, typically arising as nodular lesions that evolve into suppurative ulcers, the sporotrichotic chancre, though severe, disseminated, and central nervous system (CNS) forms have become increasingly recognized, particularly in immunocompromised or systemically vulnerable hosts (10–14).

Clinical evidence suggests that underlying diabetes *mellitus* may worsen the course of sporotrichosis (15). Despite these observations, an experimental model to systematically evaluate fungal pathogenesis and host immune responses under hyperglycemic conditions remains lacking (15).

Here, we establish and characterize a novel experimental mouse model to evaluate *S. brasiliensis* infection under type 1 diabetic conditions. Using streptozotocin-induced diabetic *C57BL/6J* mice, we observed complete mortality after infection with strain M1168, in contrast with full survival in non-diabetic controls. We comprehensively characterized the pathophysiological profile of this model by analyzing systemic fungal dissemination, inflammatory kinetics, leukocyte dynamics, macrophage functional status, and autophagic markers. This work provides a novel, robust experimental platform to study *S. brasiliensis* infection in the context of metabolic comorbidity and delineates the immunological features associated with hyper-susceptibility to severe fungal disease.

## 2 Methods

### 2.1 Animal studies

All experimental procedures were approved by the Institutional Animal Care and Use Committee (CEUA, protocol no. 642). Male wild-type *C57Bl/6J* mice (8–12 weeks old, 18–22 g) were obtained from the Animal Facility of the Faculty of Pharmaceutical Sciences, University of São Paulo (FCF-USP). Animals were housed in groups (n=5/cage) under controlled environmental conditions (22 ± 2 °C; 12/12 h light/dark cycle) with *ad libitum* access to food and water. Mice were randomly assigned to four experimental groups (**Table 1**). For each experiment, an average of 4 to 6 animals per group were used, data specifically provided in the legend for each result.

**Table 1:** Groups used during the experiment.

Experimental condition	Description		
	Groups	Day 0	Day 15
CONTROL (CT)	CT	Citrate buffer	sterile PBS
	CT + Sb	Citrate buffer	*Sporothrix brasiliensis*
DIABETIC (DT)	DT	STZ	sterile PBS
	DT + Sb	STZ	*Sporothrix brasiliensis*

The animals were divided into 4 groups and chosen at random. Sb: *Sporothrix brasiliensis*; STZ: Streptozotocin; PBS: Phosphate-buffered saline.

### 2.2 Induction of diabetes *mellitus*

Diabetes was induced by intraperitoneal (*i.p*.) injection of streptozotocin (STZ) (65 mg/kg; MCE - MedChemExpress^®^) dissolved in 0.1 M citrate buffer (pH 4.5) for five consecutive days following a 6-hour fast (6, 16, 17). Blood glucose levels were measured 15 days post-induction using a portable glucometer (Accu-Chek Advantage II; Roche Diagnostics). Animals with glycemia > 300 mg/dL (16.6 mM) were confirmed as diabetic and included in the study. For each experiment, an average of 4 to 6 animals per group were used, data specifically provided in the legend for each result.

### 2.3 Culture of *Sporothrix brasiliensis* and preparation of the inoculum

The *S. brasiliensis* M1168 strain, originally isolated from a case of meningeal sporotrichosis, was kindly provided by Prof. Dr. Sandro Rogério de Almeida (FCF-USP). Molecular analysis confirmed all isolates as *S. brasiliensis* prior to experimental use, strictly following the PCR (**Supplementary Figure 1**) protocol and primers described by Rodrigues et al. (2015) (18), using Species-specific primer sequences (**Table 2**). Fungal cultures were maintained in the yeast phase on Brain Heart Infusion (BHI) agar inside an incubator at 37 °C with weekly subcultures. For the preparation of the experimental fungal inoculum, the yeast cells were subcultured exactly 5 days prior to infection to minimize hyphal transition and ensure a predominantly viable yeast population. Yeast cells were then harvested, suspended in sterile phosphate-buffered saline (PBS), and manually counted using a Neubauer chamber (hemocytometer) with trypan blue staining prior to inoculation to determine cell viability and ensure the precise concentration of 1x10^7^ cells.

**Table 2:** Species-specific primer sequences targeting the calmodulin (*CAL*) gene in members of the *Sporothrix* genus.

Target species	Primer	Primer sequence (5´-3`)	Target	Size (bp)
*S. brasiliensis*	*Sbra-F*	CCC CCG TTT GAC GCT TGG	*CAL*	469
	*Sbra-R*	CCC GGA TAA CCG TGT GTC ATA AT		
*S. schenckii*	*Ssch-F*	TTT CGA ATG CGT TCG GCT GG	*CAL*	331
	*Ssch-R*	CTC CAG ATC ACC GTG TCA		
Internal Positive Control	*IPC-F*	ATG CGA TAC GTA ATG TGA ATT GC	5, 8S	101
	*IPC-R*	GAC GCT CGG ACA GGC ATG		

### 2.4 Subcutaneous infection and infection monitoring

*C57BL/6J* mice were subcutaneously (*s.c.*) infected in the sacral dorsal region with a standardized inoculum of 1×10^7^ viable yeast cells of *S. brasiliensis* (strain M1168) suspended in 200 µL of sterile PBS, as previously described (19). For each experiment, an average of 4 to 6 animals per group were used, with specific sample sizes provided in the respective figure legends. To monitor the course of infection over the 14-day period, the following parameters were recorded twice a week (every 3–4 days, starting on day 3 post-infection): animal survival, development of secondary lesions, body weight (g), and primary lesion diameter (cm) measured at the sole inoculation site via a digital caliper.

### 2.5 Infection monitoring and survival curve

Mice were monitored daily for clinical signs of disease, weight loss, and survival. For the survival analysis, independent cohorts of mice (n = 6 per group) were followed longitudinally up to day 31 post-infection (for the diabetic infected group) or day 36 post-infection (for the normoglycemic infected group) to record spontaneous mortality patterns. Animals that reached human endpoints (severe morbidity, loss of more than 20% of body weight, or inability to feed) were immediately euthanized using an overdose of anesthesia.

### 2.6 Collection of biological samples

In a separate experimental set designed for mechanistic and immunological analyses, mice (n = 6 per group) were monitored until the 14th day post-infection. On this specific endpoint, the animals were anesthetized (ketamine/xylazine, 300/30 mg/kg, *s.c*.) for intracardiac blood collection and euthanasia. The blood was processed to obtain plasma (EDTA) and serum (centrifugation at 8, 000 rpm/10 min). Skin lesions and organs (liver, brain, lymph node, spleen, and kidneys) were collected, weighed, and homogenized in RIPA buffer. After centrifugation, the supernatants were stored at -80 °C.

### 2.7 Total and differential cell count

Post-euthanasia, 1 mL of blood was collected via intracardiac puncture. A 45 µL aliquot was diluted in EDTA (1:10) for complete blood count (CBC) using a BC-2800 Vet automated analyzer (Mindray). The remaining blood was collected without anticoagulant, centrifuged at 8, 000 rpm for 10 min to obtain serum, and stored at -80 °C for further analysis.

### 2.8 Colony-forming unit count

Tissue samples (skin lesions, liver, brain, spleen, kidneys, and lymph nodes) were harvested, weighed, and mechanically homogenized on ice in RIPA buffer (1 mL/organ; 2 mL for liver). This extraction buffer was specifically selected to allow simultaneous high-quality total protein isolation from the same biological samples for downstream applications, thereby minimizing experimental animal usage in accordance with ethical guidelines. To determine fungal burden while strictly minimizing prolonged exposure to the buffer’s detergents, the homogenates were immediately subjected to rapid serial dilutions in sterile saline solution (0.9% NaCl). Subsequently, 100µL aliquots of the diluted samples were plated in duplicate onto Brain Heart Infusion (BHI) agar using sterile glass beads. Plates were incubated at 30°C for 7 days, and results were expressed as *log*10 colony-forming units (CFU) per gram of tissue.

### 2.9 Protein extraction and western blotting

Liver fragments were homogenized in RIPA buffer (50 mM Tris, pH 8.0; 150 mM NaCl; 1% Triton X-100; 0.1% SDS) supplemented with protease inhibitors. Total protein concentration was determined using the Pierce™ BCA Protein Assay Kit (Thermo Scientific^®^). Equal amounts of protein (40 µg) were separated by SDS-PAGE and transferred to nitrocellulose membranes. After blocking with 10% BSA, membranes were incubated with primary antibodies against autophagy markers: Beclin-1, LC3B, and SQSTM1/p62 (Cell Signaling Technology, Danvers, MA, USA). Following incubation with HRP-conjugated anti-rabbit secondary antibody, protein expression was detected. β-*actin* (Sigma-Aldrich) was used as a loading control for normalization. For each experiment, an average of 4 to 6 animals per group were used, data specifically provided in the legend for each result. See the electronic supplementary material (ESM) for further details of membranes (ESM **Figure 1**).

**Figure 1:**
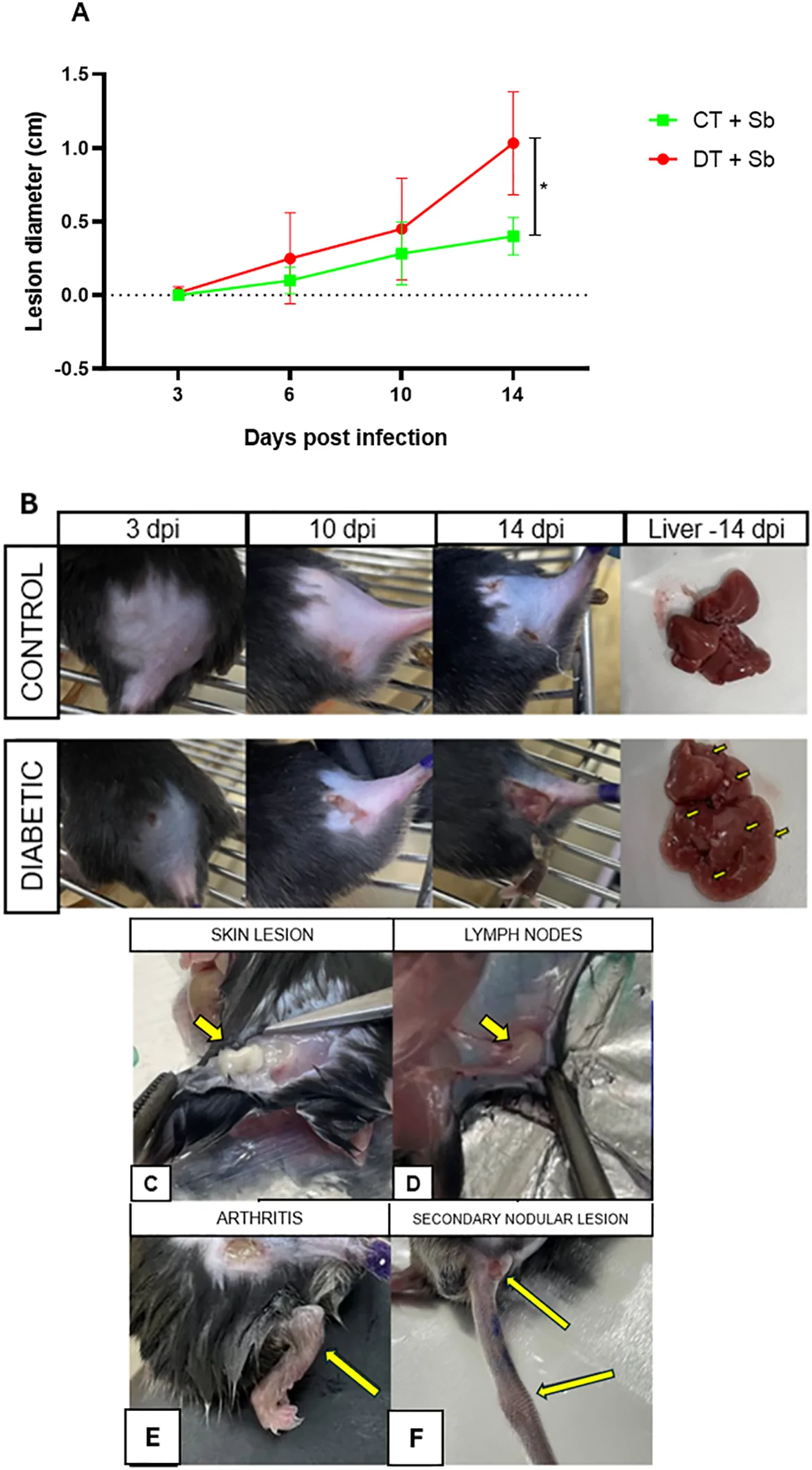
Hyperglycemia promotes uncontrolled kinetic progression of skin lesions and severe visceral dissemination. **(A)** Kinetic progression of the primary skin lesion diameter measured in centimeters (cm) over 14 days post-infection (dpi) in normoglycemic (CT+Sb) and diabetic (DT+Sb) mice. Data are expressed as mean ± SEM. Statistical significance was determined by Two-way ANOVA followed by Tukey’s multiple comparisons test; n = 6 animals per group, *p < 0.05. **(B)** Representative macroscopic pathology timeline at 3, 10, and 14 dpi, including a comparative gross inspection of the liver at 14 dpi. Note the presence of multiple metastatic visceral nodules (yellow arrows) restricted to the diabetic group. (**C–F**) Representative macroscopic hallmarks of disseminated sporotrichosis in diabetic hosts at the day 14 endpoint: **(C)** subcutaneous abscess section showing suppurative exudate (arrow); **(D)** abdominal cavity exposure highlighting regional lymphadenopathy (arrow); **(E)** severe inflammation, edema, and swelling in the paw and posterior leg joints, indicative of fungal arthritis (arrow); and **(F)** development of secondary nodular lesions distant from the primary inoculation site (arrows).

### 2.10 Immunophenotyping and flow cytometry

Spleen, lymph node, and skin lesion samples were mechanically dissociated. Splenocytes underwent osmotic lysis of erythrocytes using distilled water. Cells were counted in a Neubauer chamber and adjusted to 1x10^6^ cells/sample. For spleen and lymph node analysis, cells were stained for lymphoid markers (CD3, CD4, CD8, CD19, CD45R/B220, and IgM). Skin lesion cells were characterized using myeloid markers (MHCII/I-A[b], CD11c, CD11b, F4/80, GR1/Ly-6G, and Ly-6C), as demonstrated in **Supplementary Table 1**. To minimize non-specific Fc receptor-mediated antibody binding, cell staining and washing steps were performed in PBS supplemented with 2–3% Fetal Bovine Serum (FBS) at 4 °C. Following incubation (30 min, 4 °C), cells were washed with PBS containing 2–3% FBS. Data were acquired on a FACSCanto II flow cytometer (BD Biosciences) and analyzed using FlowJo software (BD Biosciences, Ashland, OR, USA). For each experiment, an average of 4 animals per group were used (as indicated in the figure legends). Due to channel limitations in the multicolor flow cytometry panel, a dedicated viability dye was not included. To minimize the inclusion of dead cells and non-specific antibody binding, leukocytes were isolated and processed rapidly on ice, followed by rigorous morphometric gating (*FSC-A* vs. *SSC-A*) to select intact cell populations and doublet discrimination (*FSC-A* vs. *FSC-H*). All the gates used in cytometry contain **Supplementary Material** (**Supplementary Figure 3**).

### 2.11 BMDM differentiation and phagocytosis assay

Bone marrow-derived macrophages (BMDMs) were generated from *C57BL/6J* mice 14 days post-infection. To confirm bone marrow colonization by *S. brasiliensis*, culture supernatants from isolated bone marrow cells were plated onto Brain Heart Infusion (BHI) agar for fungal growth, and species identity was definitively validated by *S. brasiliensis*-specific PCR (**Supplementary Figure 4**). Bone marrow cells were harvested from femurs and differentiated for 7 days in RPMI 1640 supplemented with 10% FBS and 30% L929-conditioned medium (LCCM) as a source of M-CSF. Because high fungal colonization in infected diabetic mice (DT+Sb) triggered severe cytotoxicity preventing successful BMDM differentiation, functional phagocytosis assays were performed using differentiated BMDMs generated from uninfected control (CT) and uninfected diabetic (DT) cohorts. LCCM was produced from L929 fibroblasts grown to 100% confluence in complete RPMI-1640 (supplemented with beta-mercaptoethanol, HEPES, and gentamicin), filtered (0.22 µm), and cryopreserved in liquid nitrogen. Prior to co-incubation, *S. brasiliensis* yeast cells (strain M1168) were pre-labeled with 25 µM CFSE for fluorescent tracking. For functional assays, BMDMs (1–2 x 10^5^ cells/well) were challenged with CFSE-pre-labeled *S. brasiliensis* at a multiplicity of infection (MOI) of 1:3 (1 macrophage to 3 fungal yeast cells). After 2 h and 4 h of co-incubation at 37 °C, cells were thoroughly washed three times with warm PBS to remove non-adherent or loosely bound extracellular yeasts. Following washing, BMDMs were stained with fluorophore-conjugated anti-CD11b and anti-F4/80 antibodies for macrophage surface identification. Data were acquired on a FACSCanto II flow cytometer to determine the percentage of fungal cell-associated macrophages (CD11b+ F4/80+ CFSE+). In the absence of a fluorescence-quenching protocol, these cytometric values represent total cell-associated fungi (encompassing both strongly adhered and internalized yeasts), maintained under strictly identical experimental conditions across all groups. Due to technical limitations regarding the number of available fluorescence channels on the flow cytometer, a viability dye was not included in the antibody panel. To minimize the inclusion of dead cells and cellular debris, a strict gating strategy based on forward scatter (*FSC-A*) and side scatter (*SSC-A*) profiles was systematically applied to select morphologically intact macrophage populations for downstream analysis.

### 2.12 Cytokine quantification

Levels of IL-2, IL-4, IL-6, IL-10, TNF-α, IFN-γ, and IL-17A were quantified in serum, tissue supernatants (liver, spleen, lymph nodes, kidney, and brain), and BMDM culture supernatants using the BD Cytometric Bead Array (CBA) Mouse Th1/Th2/Th17 Cytokine Kit (BD Biosciences, San Jose, CA, USA). Samples (50 µL) were processed according to the manufacturer’s protocol. Data acquisition was performed on a FACSCanto II flow cytometer (BD Biosciences) and analyzed with FCAP Array™ Software (BD Biosciences). Cytokine concentrations were calculated based on recombinant protein standard curves. For each experiment, an average of 4 to 6 animals per group were used, data specifically provided in the legend for each result.

### 2.13 Histopathological analysis

Liver fragments were fixed in 10% buffered formalin for 24 h, dehydrated, and embedded in paraffin according to standard protocols. Tissue sections (3 µm) were obtained and stained with Hematoxylin and Eosin (H&E) for histomorphological evaluation of tissue architecture and inflammatory infiltrates. Concurrently, sequential sections were stained with Grocott’s Methenamine Silver (GMS) to specifically confirm and characterize the presence and morphology of *Sporothrix brasiliensis* yeast structures. Slides were analyzed using a light microscope at various magnifications (20x, 40x, 60x, and 100x) to thoroughly characterize both the panoramic tissue architecture and the localized fungal elements. For each experimental parameter, 4 to 6 animals per group were analyzed, as specified in the corresponding figure legends.

### 2.14 Molecular identification by PCR

Fungal DNA was extracted from *S. schenckii*, *S. brasiliensis* (strains M1168 and 5110), and *C. albicans* suspensions via thermal lysis (95–100 °C, 15 min). PCR was performed in a 25 µL reaction volume containing 12.5 µL PCR Master Mix (Promega Corp.), 10 pmol of specific primers, and 100 ng of template DNA, targeting the *CAL* gene and the 5.8S rRNA gene as an internal positive control (18). Amplification was conducted in a Mastercycler Pro (Eppendorf) with the following cycling conditions: 95 °C for 5 min; 35 cycles of 95 °C for 1 min, 65 °C for 1 min, and 72 °C for 1 min; followed by a final extension at 72 °C for 10 min. Amplicons were resolved on a 1% agarose gel (9.5 V/cm), stained with GelRed (Biotium), and visualized using a MiniBis Pro documentation system (DNR Bio-Imaging). Specific primer sequences were used for each species, targeting the calmodulin (CAL) gene in members of the genus *Sporothrix*, as demonstrated in ESM (**Supplementary Table 2**).

### 2.15 Statistical analysis

Data were expressed as mean ± SEM and analyzed using GraphPad Prism software. Normality was assessed via the Shapiro-Wilk test. For comparisons between multiple groups, one-way or two-way analysis of variance (ANOVA) followed by Tukey’s *post-hoc* test was employed. Differences between two groups were evaluated using Student’s t-test (parametric) or the Mann-Whitney U test (non-parametric). Survival curves were analyzed using the Log-rank (Mantel-Cox) test. In all analyses, a p-value < 0.05 was considered statistically significant.

## 3 Results

### 3.1 Validation of the STZ-induced diabetic model prior to and during *Sporothrix brasiliensis* infection

To establish a reliable experimental model to characterize host-pathogen interactions under metabolic stress, we first validated the induction of type 1 diabetes *mellitus* (T1DM). Streptozotocin (STZ) induction established a homogeneous diabetic phenotype 15 days post-protocol. Experimental animals exhibited marked hyperglycemia, polydipsia, and body mass loss compared to normoglycemic controls (**Table 3**). These metabolic parameters remained consistent through day 14 post-infection (**Table 4**), confirming the stability of the STZ-induced diabetic state prior to and during fungal challenge.

**Table 3:** Characterization of the experimental model.

Protocol	Groups	Body mass (g)	Blood sugar (mg/dL)
DAY 0	CONTROL	21.63 ± 1.6*	115.69 ± 4.2*
	DIABETIC	22.99 ± 1.5	117.19 ± 6.0^§^
DAY 15	CONTROL	23.83 ± 1.8*^+^	110.25 ± 5.3^¶^
	DIABETIC	21.55 ± 1.8^+^	434.81 ± 98.3*^§ ¶^

(*) compared to the control group day 0. (+) compared to the control group day 15. (¶) compared to the control group day 15. (§) compared to the diabetic group day 0. The data represent the mean ± SEM, statistical significance was determined by the Two-way ANOVA, and multiple comparisons by Tukey’s test, n = 16 animals per group, p value < 0.05.

**Table 4:** Physiological and metabolic parameters of experimental groups.

Groups	Body mass gain (g)	Blood sugar (mg/dL)
Control (CT)	4.52 ± 0.49*	106 ± 4.1*
CT + Sb	2.18 ± 1.90^+^	112.5 ± 3.1^+^
Diabetic (DT)	-1.85 ± 4.23*	475.3 ± 118.7*^+^
DT + Sb	-3.80 ± 0.97*^+^	467.5 ± 83.7*^+^

(*) compared to the control group day. (+) compared to the control + Sb group. The data represent the mean ± SEM, statistical significance was determined by the Two-way ANOVA, and multiple comparisons by Tukey’s test, n = 6 animals per group, p value < 0.05.

### 3.2 Impact of STZ-induced diabetes on host survival, lesion progression, and *Sporothrix brasiliensis* dissemination

**Figure 2:**
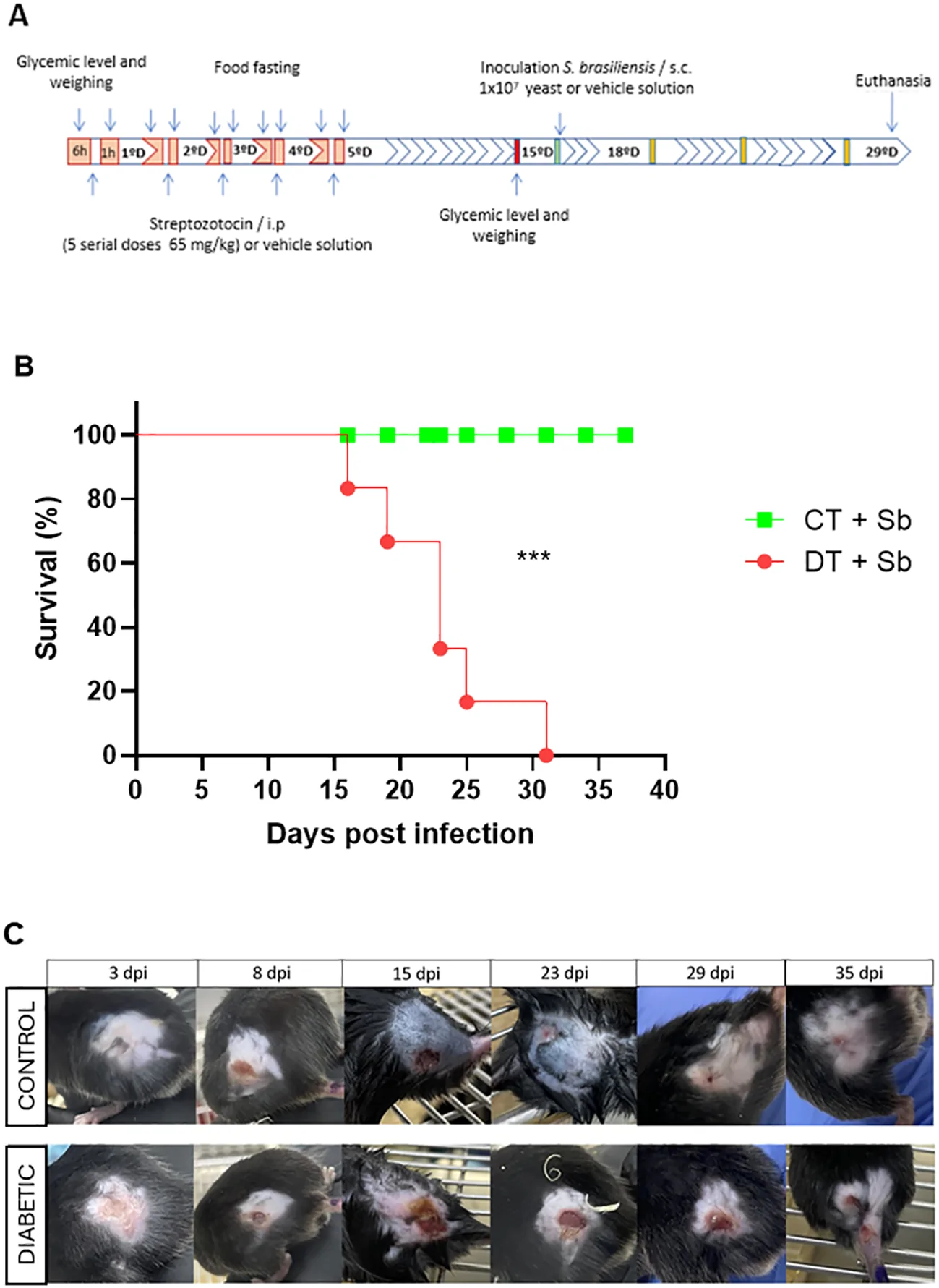
Diabetes *mellitus* exacerbates susceptibility to infection by *Sporothrix brasiliensis* and worsens lesion severity. **(A)** Experimental design timeline for the induction of type 1 diabetes *mellitus* using streptozotocin (STZ) and the subsequent co-morbidity model with *Sporothrix brasiliensis* infection. The timeline detail glycemic monitoring, weighing, food fasting, and the final infection point before euthanasia at the established endpoint. **(B)** Survival curve showing percentage of mice alive after subcutaneous inoculation with the hypervirulent *S. brasiliensis* M1168 strain. Note the 100% mortality in the diabetic infected group (DT+Sb) compared to the normoglycemic infected group (CT+Sb), which shows no spontaneous mortality. Statistical significance was determined by the Log-rank (Mantel-Cox) test. n = 6 per group, $* * * p < 0.0001$. **(C)** Representative macroscopic follow-up photographs showing the evolution of ulcerative lesions at the inoculation site taken sequentially from the same mouse within each experimental group at specific time points: 3, 8, 15, 23, 29, and 35 days post-infection (dpi) for both the Control and Diabetic groups. Note the increased severity and delayed healing in the diabetic animals compared to the control group over time.

Having confirmed the metabolic disruption in diabetic hosts, we next evaluated how this immunocompromised baseline is associated with host susceptibility and the overall phenotype of *Sporothrix brasiliensis* infection. Following the established experimental design timeline (**Figure 2A**), infected non-diabetic controls (CT+Sb) maintained 100% survival throughout the 37-day follow-up period. In contrast, the diabetic infected group (DT+Sb) exhibited a sharp decline in survival starting on day 16, reaching total mortality by day 31 (**Figure 2B**). Macroscopic monitoring demonstrated that STZ-induced diabetic mice (DT+Sb) experienced progressive clinical worsening characterized by extensive, severe ulcerative lesions arising directly at the single subcutaneous inoculation site and compromised tissue integrity, whereas the CT+Sb cohort developed milder lesions with signs of healing at later time points (**Figure 2C**).

**Figure 3:**
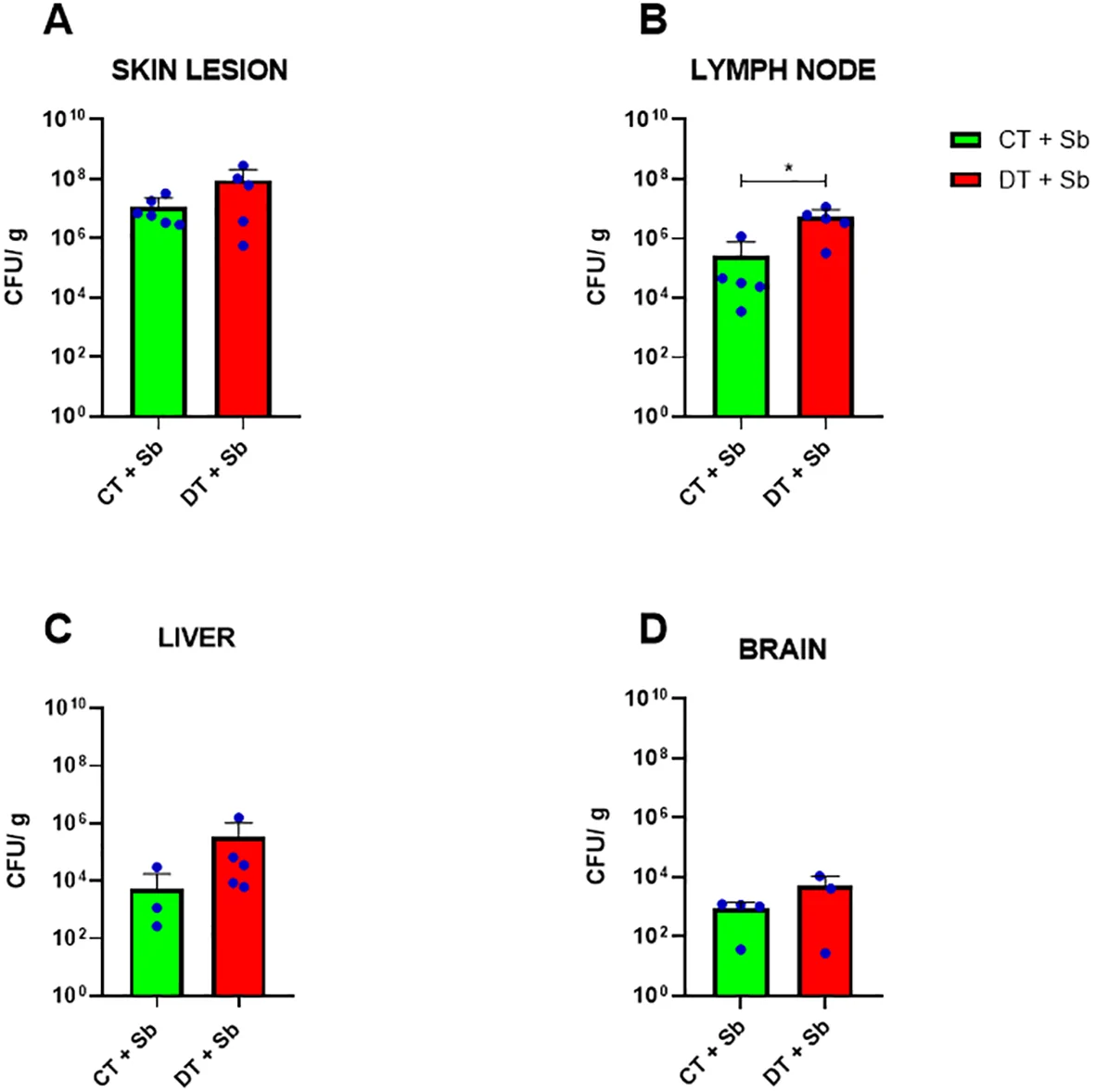
Hyperglycemia impairs regional containment of fungi in draining lymph nodes and favors systemic dissemination. **(A–D)** Fungal load quantified by colony-forming units per gram of tissue (CFU/g) on day 14 post-infection in normoglycemic (CT+Sb) and diabetic (DT+Sb) mice across different anatomical compartments: **(A)** primary skin lesion, **(B)** draining lymph node, **(C)** liver, and **(D)** brain. Data are expressed as mean ± SEM. Statistical significance was determined by the non-parametric Mann-Whitney U test; n = 6 animals per group. The asterisks indicate a statistically significant difference (*p < 0.05) restricted to the draining lymph nodes, highlighting a failure in regional fungal containment.

Consistent with the reduced survival, at 14 days post-infection, diabetic mice displayed uncontrolled wound expansion and systemic dissemination, evidenced by multifocal hepatic nodules, whereas control mice showed lesion containment and resolution (**Figures 1A, B**). While all infected animals presented encapsulated subcutaneous lesions with purulent exudate and abdominal lymphadenopathy (**Figures 1C, D**), fungal load quantification confirmed significantly higher titers in the diabetic cohort. Quantitative assessment of *Sporothrix brasiliensis* fungal burden across organs revealed a trend toward increased fungal burden in the skin, liver, and brain of DT+Sb mice (**Figure 3A, C, D**). Notably, a statistically significant increase in fungal load was observed in the draining lymph nodes of the diabetic host (p < 0.05; **Figure 3B**). Fungal burdens in kidneys and spleen remained below the limits of detection.

### 3.3 STZ-induced diabetes abrogates physiological weight gain during *Sporothrix* brasiliensis infection

To further evaluate the systemic impact of *S. brasiliensis* dissemination alongside persistent metabolic impairment, we monitored host physiological parameters throughout the infection timeline. Control groups (CT and CT+Sb) maintained normoglycemia and physiological weight gain throughout the experiment (**Table 3**). Conversely, STZ-induced mice (DT and DT+Sb) exhibited persistent hyperglycemia (>300 mg/dL) and progressive body mass loss, with the infected diabetic cohort (DT+Sb) showing the most pronounced decline by day 14 post-infection (**Table 4**), reflecting an aggravated metabolic deterioration in infected diabetic hosts.

### 3.4 Exacerbated hepatic fungal burden and granulomatous response in diabetic sporotrichosis

**Figure 4:**
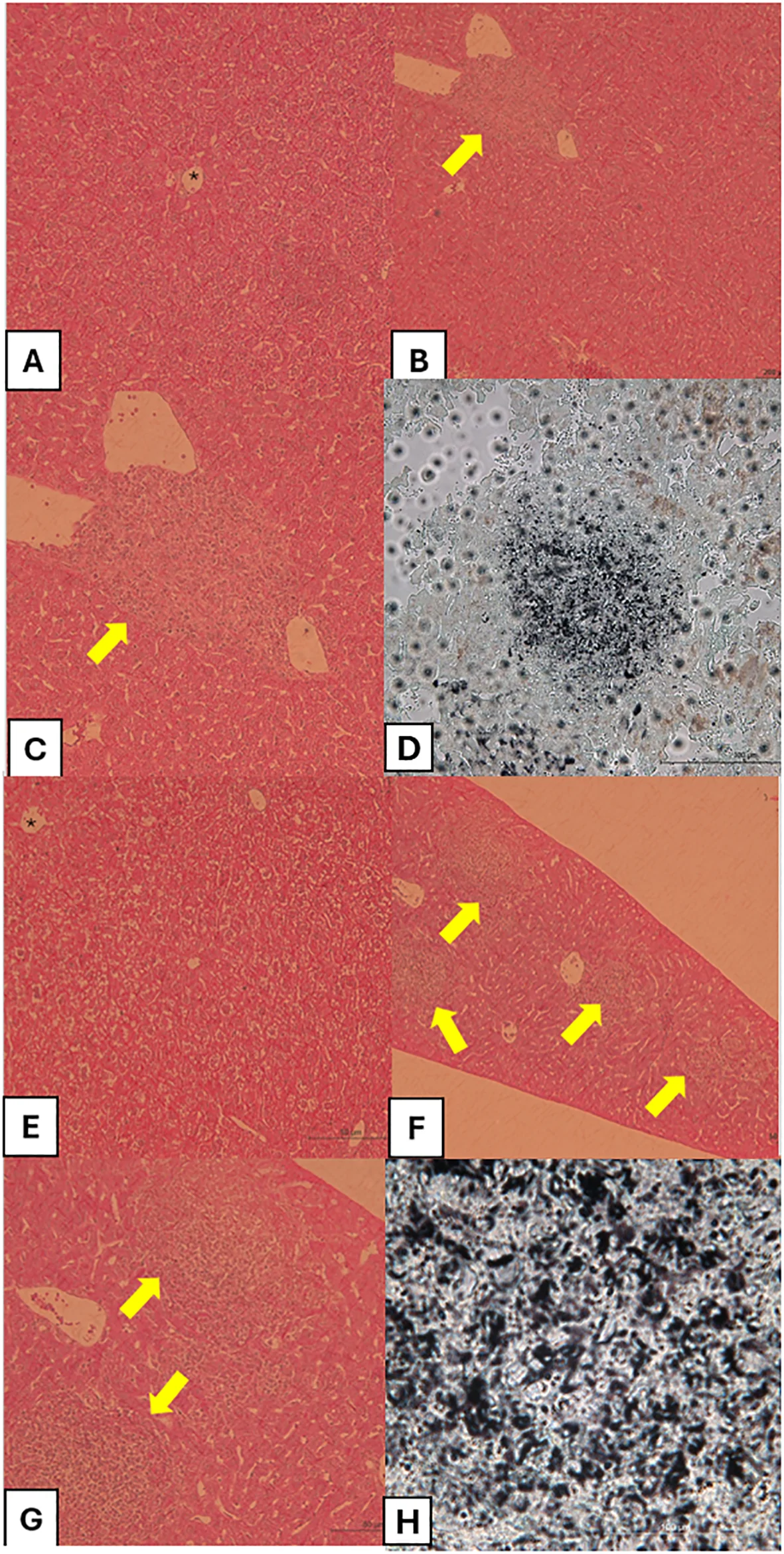
Hyperglycemia alters hepatic immunopathology and architectural response during *Sporothrix brasiliensis* infection. **(A)** Liver section from an uninfected control (CT) animal showing preserved tissue architecture. **(B)** Organised granulomatous response in the hepatic parenchyma of an infected control (CT+Sb) animal (20×). **(C)** Higher magnification (40×) detailing the well-circumscribed immune cell infiltration in the hepatic lesion shown in **(B, D)** Fungal elements clustered and tightly restricted within the host granulomatous tissue (20×). **(E)** Liver section from an uninfected diabetic (DT) animal displaying baseline tissue architecture (20×). **(F)** Diffuse, multifocal, and unorganised inflammatory lesions extensively spreading through the liver parenchyma of an infected diabetic (DT+Sb) animal (20×). **(G)** High-magnification view (40×) of a representative unrestricted inflammatory lesion in the diabetic host. **(H)** High-power view (60×) detailing a massive fungal burden and the distinctive yeast-like morphology of the pathogen proliferating freely within the tissue. (**A–C, E–G**) Hematoxylin and eosin (H&E) staining; **(D, H)** Grocott’s methenamine silver (GMS) staining. Yellow arrows indicate inflammatory and granulomatous lesions; asterisks (*) indicate the cross-section of the centrilobular vein.

Given the macroscopic evidence of visceral dissemination and increased fungal burden in diabetic hosts, we conducted histopathological evaluations to characterize organ-specific tissue damage, focusing on the liver. While uninfected groups (CT and DT) showed preserved hepatic architecture (**Figures 3A, E**), infected mice (CT+Sb and DT+Sb) developed multifocal granulomatous lesions with intense cellular infiltration and localized tissue loss (**Figures 4B–D**, **4F–H**). These pathological changes were most extensive in the diabetic cohort (DT+Sb), corroborating the marked susceptibility of diabetic hosts to restrict fungal dissemination and tissue inflammation.

### 3.5 Alterations in peripheral blood leukocyte dynamics during diabetic sporotrichosis

**Figure 5:**
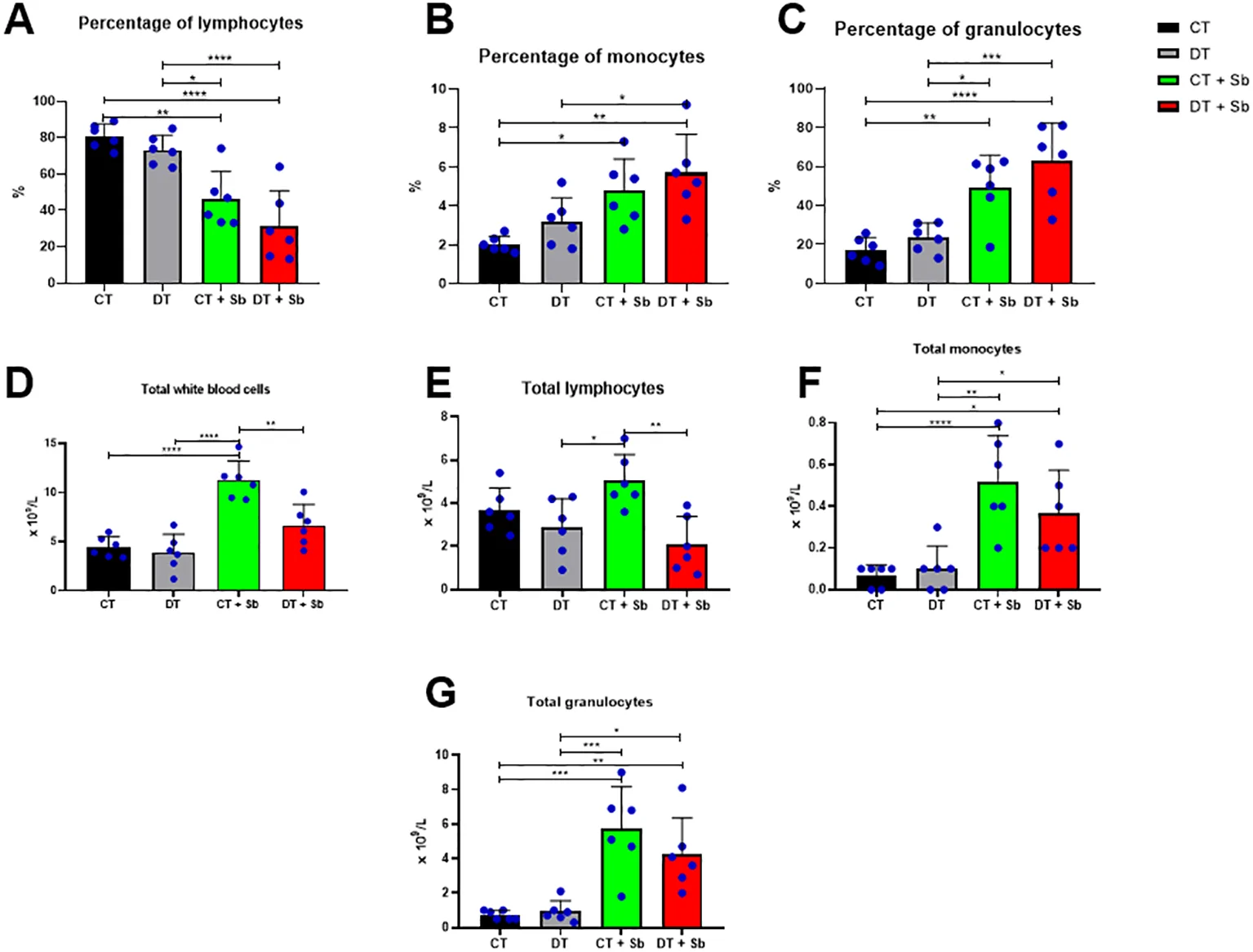
Peripheral hematological profile of healthy and diabetic mice during *Sporothrix brasiliensis* infection. **(A–C)** Relative proportion (%) of circulating leukocytes: **(A)** percentage of lymphocytes, **(B)** percentage of monocytes, and **(C)** percentage of granulocytes. (**D–G**) Absolute peripheral cell counts (x10^9^): **(D)** total white blood cell (WBC) count, **(E)** total number of lymphocytes, **(F)** total number of monocytes, and **(G)** total number of granulocytes. Experimental groups are defined as: uninfected control (CT, black bars), uninfected diabetic (DT, gray bars), normoglycemic infected (CT+Sb, green bars), and diabetic infected (DT+Sb, red bars) mice analyzed on day 14 post-infection. Data represent the mean ± SEM. Statistical significance was determined by Ordinary one-way ANOVA followed by Tukey’s multiple comparisons test; n = 6 animals per group. Asterisks indicate statistically significant differences between the linked groups, p < 0.05.

To characterize whether diabetic mice exhibited an altered systemic immune profile during infection, we evaluated the dynamics of blood leukocyte profiles during infection, focusing on comparisons between infected control (CT+Sb) and infected diabetic (DT+Sb) hosts. *S. brasiliensis* infection induced leukocytosis and a shift in cell proportions, with reduced lymphocyte percentages and increased monocytes and granulocytes (**Figures 5A–C**). In absolute terms, the expansion of total leukocytes and lymphocytes was significantly more extensive in the CT+Sb group compared to the DT+Sb cohort (**Figures 5D, E**). Although both infected groups exhibited increased circulating innate cells, diabetic hosts failed to expand their lymphocyte population. These observations suggest that the STZ-induced diabetic state is associated with blunted systemic cellular mobilization and altered lymphocyte expansion under high fungal burden.

### 3.6 Local and systemic cytokine profiling during *Sporothrix brasiliensis* infection in diabetic hosts

**Figure 6:**
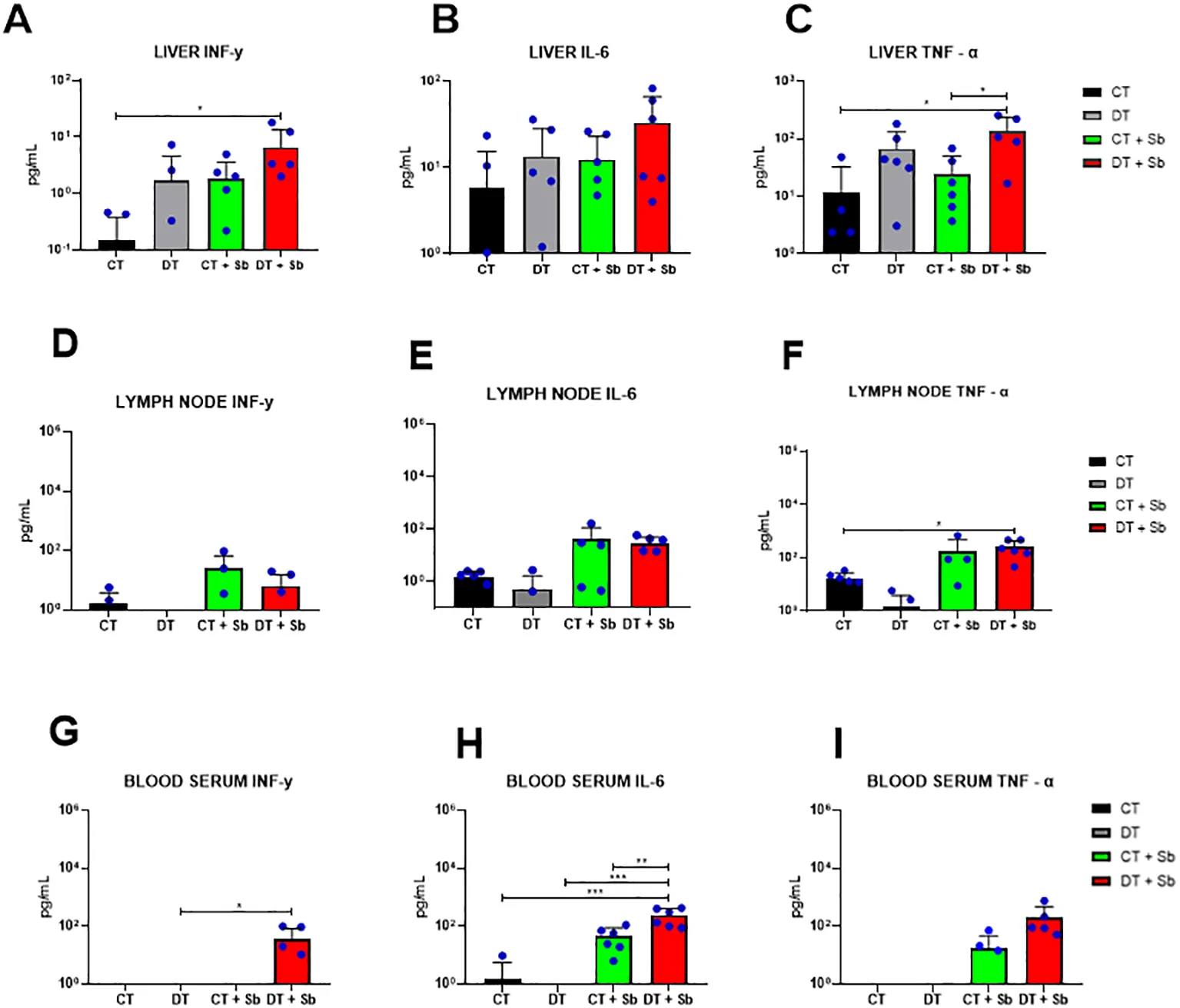
Proinflammatory cytokine profiling (IFN-γ, IL-6, and TNF-α) across hepatic tissue, draining lymph nodes, and blood serum during *Sporothrix brasiliensis* infection. Quantitative protein levels (pg/mL) of key proinflammatory cytokines were determined by Cytometric Bead Array (CBA) 14 days post-infection in healthy and diabetic mice across three biological compartments arranged in a 3x3 grid: hepatic tissue (**A–C**), draining lymph nodes (**D–F**), and blood serum (**G–I**). Evaluated cytokines include: **(A)** Liver IFN-γ, **(B)** Liver IL-6, **(C)** Liver TNF-α, **(D)** Lymph Node IFN-γ, **(E)** Lymph Node IL-6, **(F)** Lymph Node TNF-α, **(G)** Blood Serum IFN-γ, **(H)** Blood Serum IL-6, and **(I)** Blood Serum TNF-α. Experimental groups are defined as: uninfected control (CT, black bars), uninfected diabetic (DT, gray bars), normoglycemic infected (CT+Sb, green bars), and diabetic infected (DT+Sb, red bars). Data are expressed as mean ± SEM (n = 4–6 animals per group). Statistical significance was determined by Two-way ANOVA followed by Tukey’s multiple comparisons test; asterisks indicate statistically significant differences between the linked groups (p < 0.05). Cytokines showing no statistically significant differences across compartments (IL-17A, IL-10, IL-4, and IL-2) are provided in **Supplementary Information** (**Supplementary Figure 5**).

To characterize key inflammatory mediators associated with host susceptibility, we profiled the core proinflammatory cytokine panel (IFN-γ, IL-6, and TNF-α) across three distinct compartments: liver, draining lymph nodes, and blood serum (**Figures 6A–I**). In hepatic tissue, DT+Sb mice exhibited a significant increase in IFN-γ compared to CT controls (**Figure 6A**; p < 0.05), alongside a marked elevation in TNF-α relative to both CT and CT+Sb groups (**Figure 6C**; p < 0.05). Hepatic IL-6 levels showed a higher mean trend in DT+Sb mice without reaching statistical significance (**Figure 6B**). In draining lymph nodes, TNF-α was significantly elevated in DT+Sb mice compared to CT controls (**Figure 6F**; p < 0.05), while IFN-γ (**Figure 6D**) and IL-6 (**Figure 6E**) showed comparable levels between infected cohorts.

Systemically, in blood serum, DT+Sb mice displayed significantly higher IFN-γ levels compared to uninfected DT controls (**Figure 6G**; p < 0.05), as well as significantly elevated IL-6 levels compared to all other experimental groups (**Figure 6H**; p < 0.05). Serum TNF-α displayed an elevated trend in infected cohorts without statistical divergence between CT+Sb and DT+Sb (**Figure 6I**).

Cytokines that did not display statistically significant differences across experimental groups (including local and systemic levels of IL-2, IL-4, IL-10, and IL-17A) are provided in the Electronic Supplementary Material (**Supplementary Figure 5**). Altogether, these data indicate that *S. brasiliensis* infection under STZ-induced diabetic conditions corresponds to a selective proinflammatory profile characterized by elevations of IL-6, TNF-α, and IFN-γ in blood serum and affected tissues.

### 3.7 Immunophenotypic characterization of effector leukocytes during diabetic sporotrichosis

To further characterize the leukocyte subsets within this altered immune profile, we performed immunophenotypic characterization of effector lymphocytes and myeloid populations in peripheral lymphoid tissues (spleen and lymph nodes) as well as within the primary cutaneous infection site.

**Figure 7:**
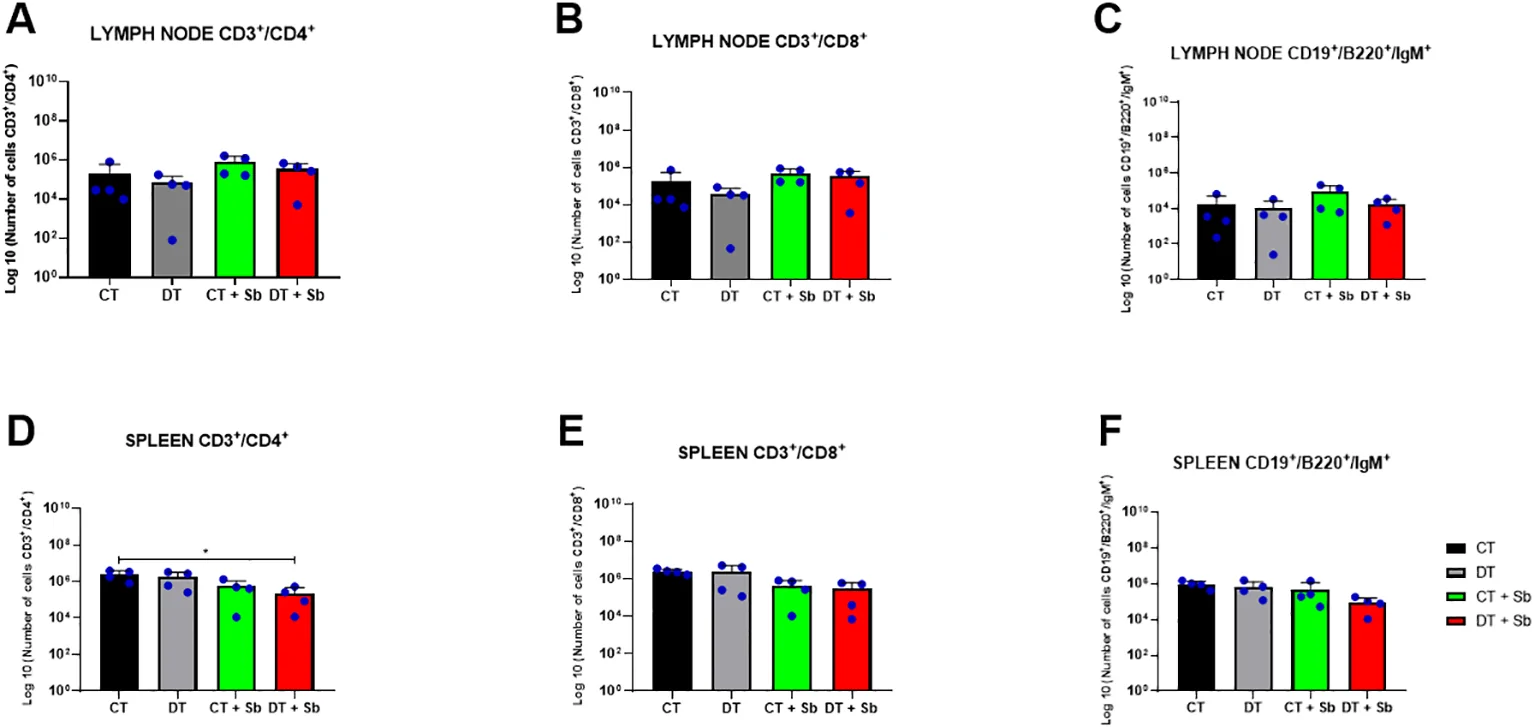
Immunophenotyping profile of T and B lymphocyte populations in lymph nodes and spleen assessed by flow cytometry. **(A–C)** Lymph node cellularity: **(A)** T helper lymphocytes (CD3^+^/CD4^+^), **(B)** cytotoxic T lymphocytes (CD3^+/^CD8^+^), and **(C)** B lymphocytes (CD19^+^/B220^+^/IgM^+^). (**D–F**) Splenic cellularity: **(D)** T helper lymphocytes (CD3^+^/CD4^+^), **(E)** cytotoxic T lymphocytes (CD3^+^/CD8^+^), and **(F)** B lymphocytes (CD19^+^/B220^+^/IgM^+^). Data represent absolute cell numbers (l*og*10 scale) expressed as mean ± SEM. Experimental groups: uninfected control (CT, black bars), uninfected diabetic (DT, gray bars), normoglycemic infected (CT+Sb, green bars), and diabetic infected (DT+Sb, red bars). Statistical significance was determined by Ordinary one-way ANOVA followed by Tukey’s multiple comparisons test; n = 4 animals per group. The asterisk indicates a statistically significant difference (p < 0.05) observed only in splenic T helper lymphocytes.

Analysis of lymphocyte populations revealed a significant reduction in the frequency of CD4+ T cells in the spleen of the DT+Sb group compared to controls (**Figure 7D**; p < 0.05). No statistical differences were observed in other T or B cell populations across the spleen or lymph nodes (**Figures 7A–C, E, F**).

**Figure 8:**
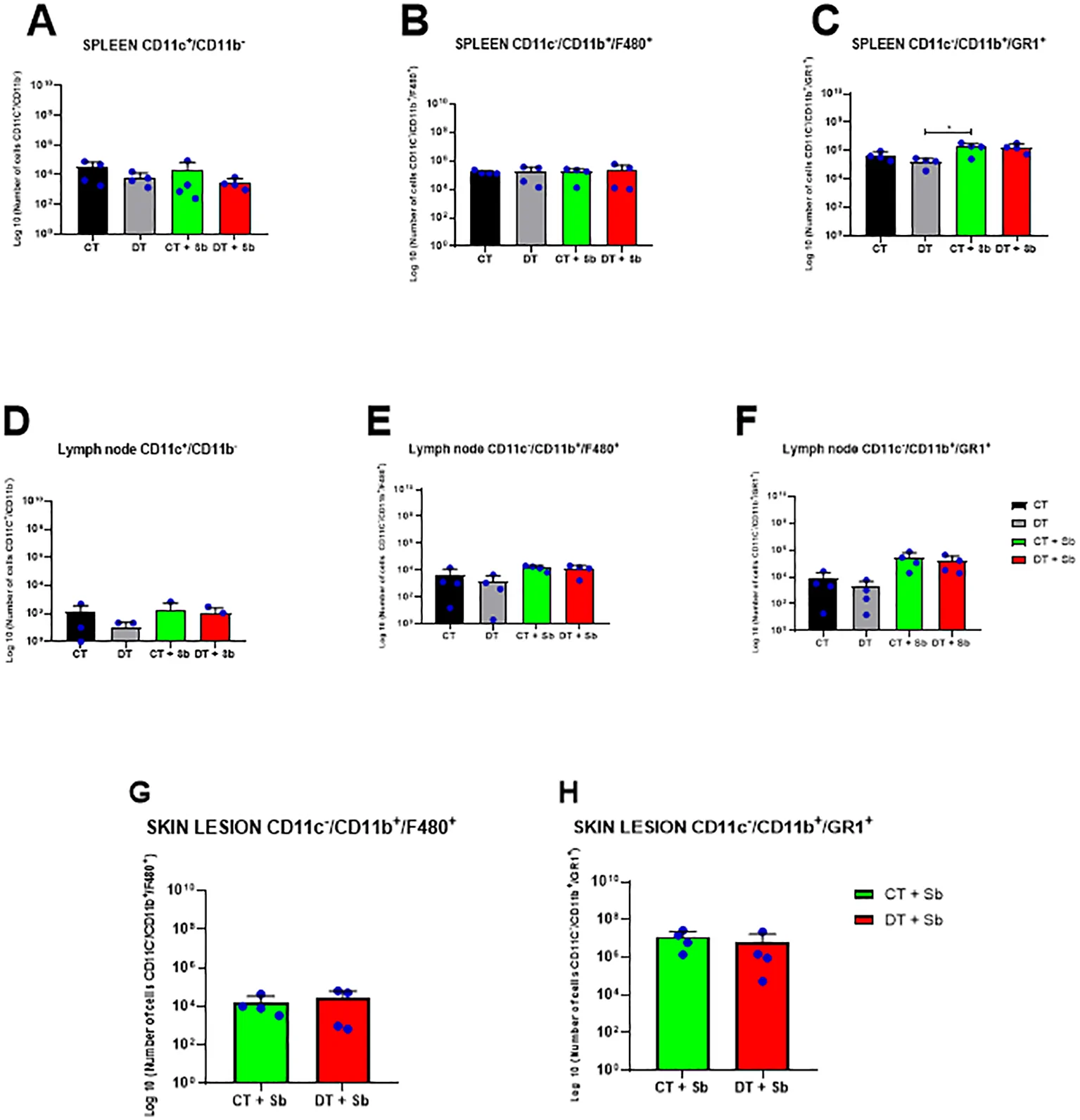
Characterization of myeloid cell populations in the spleen, draining lymph nodes, and primary skin lesions assessed by flow cytometry. **(A–C)** Splenic myeloid populations: **(A)** dendritic cells (CD11c^+^/CD11b^-^), **(B)** macrophages (CD11c^-^/CD11b^+^/F4/80^+^), and **(C)** neutrophils (CD11c^-^/CD11b^+^/Gr^+^). (**D–F**) Draining lymph node myeloid populations: **(D)** dendritic cells, **(E)** macrophages, and **(F)** neutrophils. (**G–H**) Primary skin lesion cellular infiltrate: **(G)** macrophages (CD11c^-^/CD11b^+^/F4/80^+^) and **(H)** infiltrating neutrophils (CD11c^-^/CD11b^+^/Gr1^+^). Data represent absolute cell numbers (*log*10 scale) expressed as mean ± SEM (n = 4 animals per group). For the four-group comparisons (**A–F**), statistical significance was determined by Ordinary one-way ANOVA followed by Tukey’s multiple comparisons test; the asterisk indicates a statistically significant difference (p < 0.05) between the linked groups in panel **(C)** For the two-group skin lesion comparisons (**G, H**), statistical significance was evaluated using the Unpaired t-test (or Mann-Whitney U test), showing no significant differences (p > 0.05). Experimental groups: uninfected control (CT, black bars), uninfected diabetic (DT, gray bars), normoglycemic infected (CT+Sb, green bars), and diabetic infected (DT+Sb, red bars).

Regarding innate immunity, the infected control group (CT+Sb) exhibited a significantly higher frequency of splenic neutrophils compared to the DT group (**Figure 8C**). At the primary site of infection, characterization of cutaneous lesions demonstrated local infiltration of innate effector cells, with neutrophils and macrophages quantified directly within the skin tissue (**Figures 8G, H**). However, these myeloid populations, including dendritic cells and macrophages, showed no statistically significant variations across the skin or other evaluated tissues (**Figures 8A, B, D–H**). Taken together, these findings point toward selective alterations in CD4+ T cell frequencies and systemic leukocyte dynamics in diabetic hosts, rather than an impairment in initial innate cell migration to the primary skin lesion.

### 3.8 Kinetics of *S. brasiliensis* phagocytosis by diabetic macrophages and bone marrow cytotoxicity

**Figure 9:**
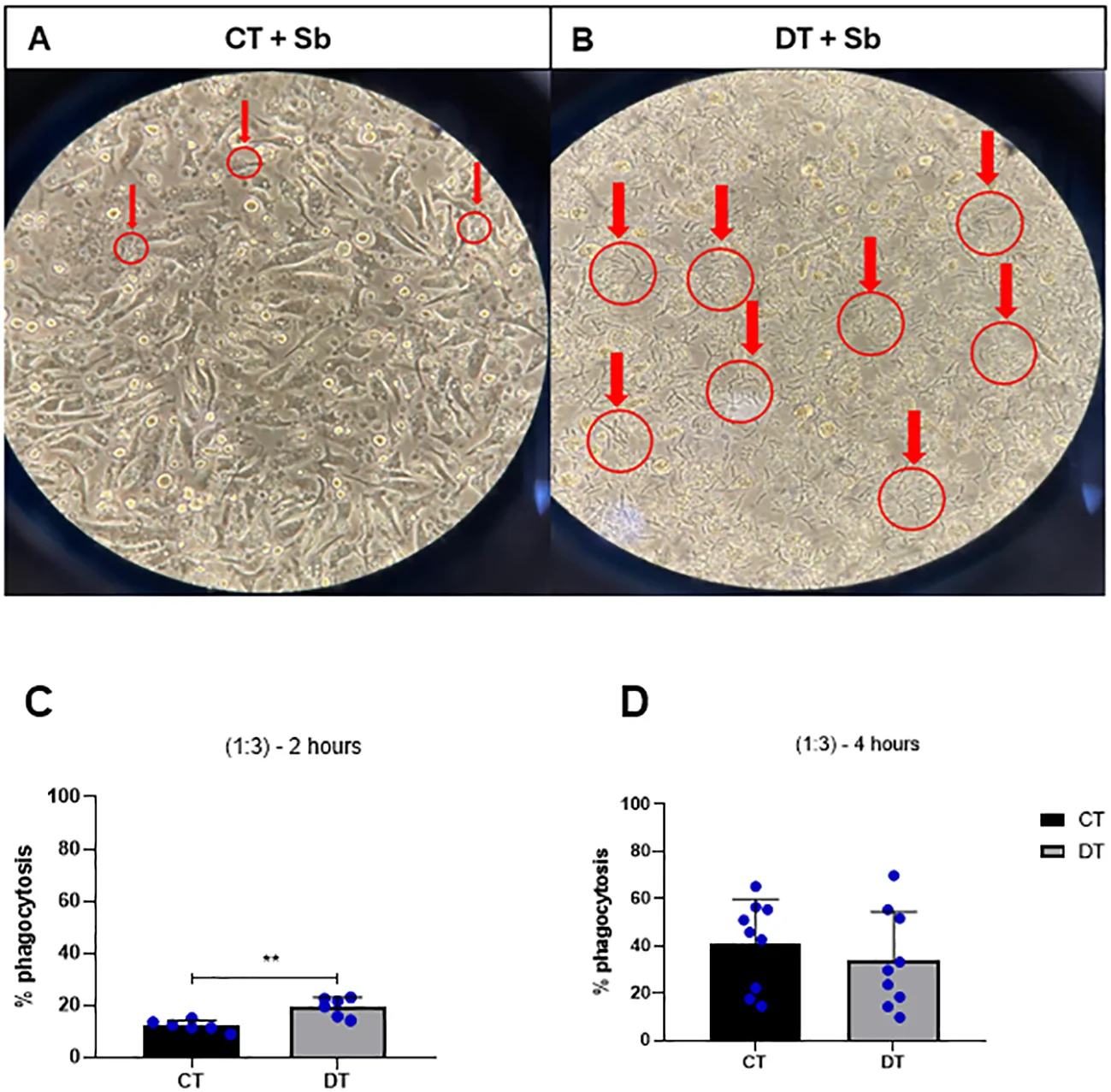
Isolation, characterization, and functional phagocytosis assay of bone marrow-derived macrophages (BMDMs). **(A, B)** Representative phase-contrast microscopy images of primary bone marrow cell cultures from infected experimental groups at day 14 post-infection: **(A)** CT+Sb cohort showing intact macrophage monolayers with localized yeast forms (red circles and arrows); and **(B)** DT+Sb cohort highlighting extensive fungal proliferation and hyphae development throughout the plate (red circles and arrows). (**C, D**) Representative flow cytometry contour plots displaying cell size (*FSC-A*) versus granularity (*SSC-A*) profiles used for characterization and gating strategies of BMDMs obtained from uninfected control [**(C)**, CT] and uninfected diabetic [**(D)**, DT] mice. Data represent the mean ± SEM from n = 6 independent samples per group. Statistical significance was determined using the non-parametric Mann-Whitney U test. Asterisks indicate a statistically significant difference (p < 0.05).

Because macrophages serve as primary effector cells in controlling fungal infections, we sought to evaluate macrophage functional responses within this model. BMDM differentiation from infected animals (CT+Sb and DT+Sb) was inhibited by the massive presence of *S. brasiliensis* yeasts and hyphae within the bone marrow, compromising cell recovery before maturation (**Figures 9A, B**). Consequently, functional assays were restricted to macrophages derived from the uninfected groups (CT and DT). Differentiated BMDMs were confirmed and gating-characterized by surface expression of specific macrophage markers (CD11b^+^F4/80^+^) via flow cytometry prior to phagocytic assays. Phagocytosis assays (1:3 ratio) revealed distinct temporal dynamics: at 2 hours, DT macrophages showed a significantly higher phagocytic percentage than CT cells (**Figure 9C**; p < 0.05). However, by 4 hours, this initial kinetic difference subsided, and no statistically significant differences in phagocytic rates were observed between the groups (**Figure 9D**), suggesting that initial fungal uptake remains functional, but downstream intracellular processing may be altered.

### 3.9 Evaluation of hepatic autophagic flux during *Sporothrix brasiliensis* infection under diabetic conditions

Given that effective fungal clearance is often coordinated with intracellular degradative pathways such as autophagy, we evaluated three key autophagic markers in the liver to assess sequential stages of the autophagic pathway: Beclin-1, an essential initiator of autophagosome nucleation; LC3B-II, a membrane-bound marker involved in autophagosome elongation and closure; and sequestosome-1 (SQSTM1/p62), a selective cargo adaptor protein degraded alongside autophagic contents upon fusion with lysosomes (20). Evaluating these markers allows for the differentiation between early pathway activation and late-stage degradative flux. Chronic diabetic metabolic stress is increasingly recognized to disturb autophagic machinery, frequently causing late-stage autophagic blockage and impaired lysosomal clearance (21).

**Figure 10:**
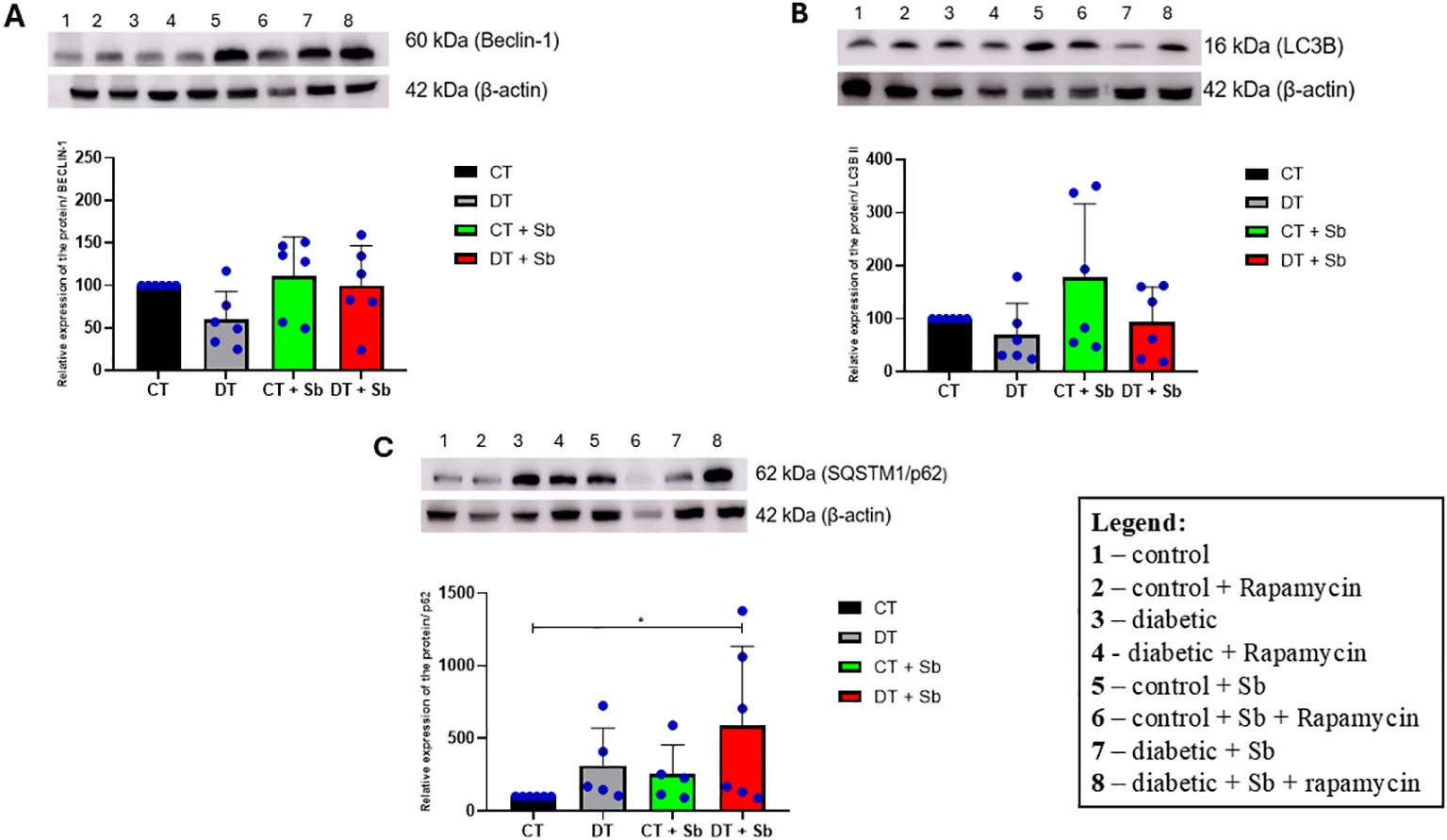
Relative expression of autophagy pathway proteins in hepatic tissue analyzed 14 days post-infection by *Western blotting*. Quantitative densitometric analysis and representative immunoblots for autophagy-related targets: **(A)** Beclin-1 (60 kDa), **(B)** LC3B-II (16 kDa), and **(C)** SQSTM1/p62 (62 kDa), with beta-*actin* (42 kDa) serving as the internal loading control. Graphical data represent relative protein expression normalized to control levels and are expressed as mean ± SEM (n = 5–6 animals per group). Experimental groups quantified in graphs: uninfected control (CT, black bars), uninfected diabetic (DT, gray bars), normoglycemic infected (CT+Sb, green bars), and diabetic infected (DT+Sb, red bars). Statistical significance was determined by Ordinary one-way ANOVA followed by Tukey’s multiple comparisons test; the asterisk indicates a statistically significant difference (p < 0.05). Representative blot panels display all 8 continuous lanes from the original uncropped membranes to preserve raw data integrity, numbered and defined in the figure legend box (Lanes 1–8). Cohorts treated with Rapamycin (Lanes 2, 4, 6, and 8) were run on the same dynamic gel for screening protocols but were excluded from the final graphical analysis as they fall outside the specific scope of the primary pathological comparisons addressed in this manuscript.

Analysis of hepatic autophagy markers showed no significant variations in Beclin-1 expression across the experimental groups (**Figure 10A**). Similarly, LC3B-II levels exhibited a non-significant trend toward an increase in the infected control group (CT+Sb) compared to the other cohorts (**Figure 10B**). However, SQSTM1/p62 expression was significantly elevated in the infected diabetic group (DT+Sb) compared to the uninfected control group (CT) (**Figure 10C**; p < 0.05). Because p62 is normally degraded during complete autophagic turnover, its significant accumulation indicates a failure in downstream autolysosomal degradation, suggesting an association between the STZ-induced diabetic state and altered late-stage autophagic markers during *S. brasiliensis* infection.

## 4 Discussion

Establishment of sustained hyperglycaemia before inoculation with *S. brasiliensis* allowed the immune responses analyzed here to be interpreted as consequences of the diabetic metabolic state rather than acute metabolic fluctuation, thereby more closely modelling the immune dysfunction observed in patients with diabetes (4–6, 16, 22). Within the *Sporothrix* complex, *S. brasiliensis* is regarded as the most virulent species, associated with enhanced tissue invasion, increased fungal burden, and severe clinical disease (23–25), including neurotropism and CNS involvement (12, 14, 25), as well as the zoonotic epidemic sustained by infected cats in Brazil (10, 11). Against this background, our study establishes and characterizes a novel experimental mouse model that provides a robust framework to evaluate how diabetic metabolic disruption is associated with host defense alterations against a highly virulent strain.

Although *C57BL/6J* and *BALB/c* mice generally show high survival after experimental infection with *Sporothrix* spp (24, 26)., diabetic mice infected with the M1168 strain exhibited rapid, total mortality, contrasting with full survival in normoglycemic controls. This striking divergence demonstrates that the diabetic metabolic milieu profoundly alters the phenotypic progression of host defense and fungal interaction. To avoid survival bias and peri-mortem tissue degradation, day 14 post-infection was selected for mechanistic, cellular, and immunological analyses. The M1168 strain was originally isolated from a case of meningeal sporotrichosis, and its behavior in our model is consistent with an intrinsically aggressive phenotype. The exacerbated cutaneous ulceration, regional lymphadenopathy, and systemic dissemination observed in STZ-induced diabetic hosts indicate that diabetes is associated with reduced early pathogen containment and enhanced systemic fungal presence (19, 27).

This phenotype is reinforced by the pronounced worsening of cutaneous disease in diabetic mice. As the diabetic metabolic state compromises tissue repair via hypoxia, oxidative stress, and dysregulated cellular recruitment (22), the exacerbated lesion progression and purulent exudate indicate that STZ-induced diabetes is accompanied by heightened local tissue injury and delayed reparative responses, concurring with systemic spread.

Body mass trajectories further support systemic metabolic decompensation. The accentuated weight loss in infected diabetic hosts reflects an aggravated catabolic state associated with insulin deficiency and increased metabolic demands (6, 16, 17, 28). Thus, the severe lethal outcome reflects a complex interaction between a highly virulent fungal infection and pre-existing metabolic destabilization.

In agreement with clinical reports linking diabetes to severe, disseminated sporotrichosis (15, 29–34), our experimental model confirms that STZ-induced diabetes corresponds to a more aggressive, systemic phenotype of *S. brasiliensis* infection. In particular, the recovery of viable fungi from brain tissue in this model aligns with the neurotropic potential of the M1168 strain, though systemic dissemination in diabetic hosts was most significantly pronounced in draining lymph nodes rather than reflecting a selective exacerbation of cerebral fungal load.

The organ tropism of M1168 further highlights this altered disease pattern. Unlike *S. brasiliensis* ATCC 5110, which colonizes the liver, spleen, and kidneys (35, 36), M1168 exhibited preferential recovery from the liver, lymph nodes, and brain. Hepatic dissemination was accompanied by gross nodules and microscopic granulomatous lesions (23, 27, 36), which were strikingly more extensive in diabetic hosts (27, 33, 37). Although granuloma formation normally restricts fungal spread (38) and imposes metabolic restraint on pathogens (39), under diabetic conditions this structure appears morphologically prominent yet functionally insufficient to fully prevent tissue damage.

The immune phenotype in diabetic infected mice was characterized by exaggerated inflammatory signaling coupled with altered effector cellular profiles. While diabetic hosts exhibited elevated levels of key proinflammatory mediators such as TNF-α, IFN-γ, and IL-6 (27), this inflammatory profile did not correspond to host protection. This failure is underscored by the absence of compensatory increases in regulatory IL-10 or induction of IL-17, the latter being vital for neutrophil-mediated antifungal tissue defense (40, 41). Without balanced counter-regulatory control, the persistent TNF-α/IL-6 axis correlates with tissue hyperinflammation rather than a protective, coordinated Th1/Th17 profile.

Furthermore, in the diabetic host, this response presented as a dysregulated inflammatory state coexisting with impaired fungal restriction. This interpretation is further supported by the attenuated systemic leukocytic mobilization in the DT+Sb group compared with infected controls and by the significant reduction in splenic CD4^+^ T cells. Rather than a mere passive alteration in lymphoid cellularity, this marked reduction in CD4+ frequencies likely reflects an impairment in host cellular expansion, alterations in lymphocyte trafficking, or functional exhaustion associated with high fungal burden and persistent diabetic stress (42). Under persistent STZ-induced diabetic conditions, this altered immune baseline is amplified, compromising the coordination of a protective T-cell response. Thus, diabetes generates a paradoxical state in which inflammatory activation is preserved or amplified, whereas the coordinated protective cellular response is compromised, a landscape in which innate immune activation fails to effectively prime or maintain adaptive control over the pathogen. The steady lymphocyte cellularity observed in draining lymph nodes at day 14 post-infection likely reflects a kinetic equilibrium, wherein initial lymph node cell expansion gives way to continuous effector lymphocyte efflux toward inflamed peripheral tissues, such as the primary cutaneous lesion.

Furthermore, while specific intracellular Th17 immunophenotyping was not directly performed, evaluation of systemic and local IL-17A levels revealed low, non-significant cytokine induction across groups (**Supplementary Figure 5**), indicating an absence of robust IL-17A upregulation during infection in this model.

Bone marrow involvement represents another critical aspect of this systemic pathology. Our detection of *S. brasiliensis* within the bone marrow niche suggests that this compartment can be colonized under impaired host defense (43, 44), potentially influencing hematopoietic output and effector cell supply during advanced infection.

Our *in vitro* data further indicate that macrophage functional alterations represent a key feature of this phenotype. *S. brasiliensis* exhibits greater cytotoxicity towards host macrophages than *S. schenckii*, linked to rapid intracellular proliferation and cell death (45). In this context, diabetic macrophages displayed a transient early increase in fungal uptake at 2 h, which rapidly declined by 4 h compared to control cells. Rather than indicating superior clearance, this early burst likely reflects accelerated fungal internalization leading to premature cellular overload, metabolic stress, and reduced viability in diabetic cells (46, 47).

To evaluate potential cellular features accompanying this phagocytic failure, we evaluated key autophagic flux markers (20, 48, 49). While Beclin-1 and LC3B-II levels remained unchanged, indicating intact initial autophagosome formation (20, 50, 51), p62 (SQSTM1) accumulated significantly in infected diabetic hosts. Because p62 is an autophagic substrate degraded upon completion of the autophagic process, its accumulation suggests a potential alteration in late-stage autophagic clearance (20), resembling defects observed in pancreatic beta cells in type 2 diabetes (52). Furthermore, *S. brasiliensis* evasion strategies are known to interact with this pathway: cell wall melanin masks PAMPs (such as beta-glucans), scavenges ROS, and inhibits NADPH oxidase, thereby blocking LC3-associated phagocytosis (LAP) recruitment (53) and altering TLR2-dependent clearance (54). In a diabetic host already predisposed to inflammatory dysregulation (55), this interaction correlates with incomplete autophagic degradation, intracellular fungal persistence, and cellular stress.

Finally, we acknowledge that the association between altered autophagic markers and increased *S. brasiliensis* burden in diabetic mice remains largely correlative at this stage. In static *Western blot* analyses without *in vivo* lysosomal flux inhibitors, elevated p62 levels primarily reflect substrate accumulation rather than direct kinetic flux blockage. Future studies utilizing pharmacological inducers (such as rapamycin) or inhibitors are warranted to establish whether restoring autophagic flux directly enhances fungal clearance in T1DM hosts.

While this STZ-induced model does not fully encompass all features of human diabetes, and direct measurements of blood-brain barrier permeability or dynamic autophagic flux were not performed, the integration of survival, histopathology, immune profiling, and cellular assays provides a robust experimental platform. Additionally, regarding immunophenotyping, although strict morphometric gating and rapid processing on ice were implemented, cell staining and washing steps relied on FBS-supplemented PBS without a dedicated viability dye or specific Fc block. Consequently, minor immunophenotypic variations across groups might have been attenuated by background noise, suggesting that more pronounced or discrete leukocytic differences could emerge in future studies utilizing refined flow cytometry panels with explicit viability markers and Fc receptor blockers. Overall, our data characterize a severe disease profile in which diabetes accompanies the transformation of *S. brasiliensis* infection from a contained mycosis into a lethal disseminated condition, marked by dysregulated inflammatory control, altered phagocyte performance, and p62 accumulation.

## 5 Conclusion

In conclusion, this study successfully establishes and characterizes a novel experimental model of *Sporothrix brasiliensis* infection under type 1 diabetic conditions. Diabetic mice exhibited pronounced hyper-susceptibility to infection, accompanied by uncontrolled fungal dissemination, severe tissue pathology, dysregulated inflammatory kinetics, altered macrophage kinetics, and hepatic p62 accumulation. By delineating the clinicopathological and immunometabolic phenotype of this model, our findings provide a valuable experimental platform for future investigations into fungal pathogenesis, comorbidity interactions, and targeted therapeutic strategies for severe sporotrichosis.

## Data Availability

The raw data supporting the conclusions of this article will be made available by the authors, without undue reservation.

## References

[1] International Diabetes Federation . IDF Diabetes Atlas, 11th Edition. Brussels, Belgium: International Diabetes Federation (2025). Available online at: https://diabetesatlas.org/media/uploads/sites/3/2025/04/IDF_Atlas_11th_Edition_2025.pdf (Accessed June 7, 2026).

[2] Coelho FR Martins JO . Diagnostic methods in sepsis: The need of speed. Mag Braz Med Assoc. (2012) 58:498–504. doi: 10.1590/S0104-42302010000400024 22930032

[3] Anjos-Valotta EA Martins JO Oliveira MA Casolari DA Britto LR Tostes RC . Inhibition of tumor necrosis factor-alpha-induced intercellular adhesion molecule-1 expression in diabetic rats: Role of insulin. Inflammation Res. (2006) 55:16–22. doi: 10.1007/s00011-005-0003-7 16328103

[4] Galicia-Garcia U Benito-Vicente A Jebari S Larrea-Sebal A Siddiqi H Uribe KB . Pathophysiology of type 2 diabetes *mellitus*. Int Mol Sci. (2020) 21:6275. doi: 10.3390/ijms21176275 32872570 PMC7503727

[5] American Diabetes Association . Classification and diagnosis of diabetes: Standards of medical care in diabetes—2018. Diabetes Care. (2018) 41:S13–27. doi: 10.2337/dc18-S002 29222373

[6] Guimarães JPT Filgueiras LR Martins JO Jancar S . Leukotriene involvement in the insulin receptor pathway and macrophage profiles in muscles from type 1 diabetic mice. Mediat Inflammation. (2019) 2019:1–8. doi: 10.1155/2019/4596127 30809106 PMC6369485

[7] Oliveira MM Almeida-Paes R Gutiérrez-Galhardo MC Zancope-Oliveira RM . Molecular identification of the *Sporothrix schenckii* complex. Rev Iberoam Micol. (2014) 31:2–6. doi: 10.1016/j.riam.2013.09.008 24270070

[8] Qin J Zhang J . Sporotrichosis. N Engl J Med. (2019) 380:771. doi: 10.1111/j.1365-4362.1986.tb02221.x 30786190

[9] Howard DH . Dimorphism of sporotrichum schenckii. J Bacteriol. (1961) 81:464–9. doi: 10.1128/jb.81.3.464-469.1961 13716207 PMC279030

[10] Morgado DS Castro R Ribeiro-Alves M Corrêa-Moreira D Castro-Alves J Pereira AS . Global distribution of animal sporotrichosis: A systematic review of *Sporothrix sp*. identified using molecular tools. Curr Res Microb Sci. (2022) 3:100140. doi: 10.1016/j.crmicr.2022.100140 35909607 PMC9325896

[11] Montenegro H Rodrigues AM Galvão Dias MA Da Silva EA Bernardi F Camargo ZP . Feline sporotrichosis due to *Sporothrix brasiliensis*: An emerging animal infection in São Paulo, Brazil. BMC Vet Res. (2014) 10:269. doi: 10.1186/s12917-014-0269-5 25407096 PMC4244058

[12] Mialski R Oliveira JN Jr Silva LH Kono A Pinheiro RL Teixeira MJ . Chronic meningitis and hydrocephalus due to *Sporothrix brasiliensis* in immunocompetent adults: A challenging entity. Open Forum Infect Dis. (2018) 5:ofy081. doi: 10.1093/ofid/ofy081 29977951 PMC6007373

[13] Queiroz-Telles F Buccheri R Benard G . Sporotrichosis in immunocompromised hosts. J Fungi. (2019) 5:8. doi: 10.3390/jof5010008 30641918 PMC6463096

[14] Lima MA Freitas DFS Oliveira RVC Fichman V Varon AG Freitas AD . Meningeal sporotrichosis due to *Sporothrix brasiliensis*: A 21-year cohort study from a Brazilian reference center. J Fungi. (2022) 9:17. doi: 10.3390/jof9010017 36675837 PMC9863964

[15] Oliveira MA De Almeida SR Martins JO . Novel insights into sporotrichosis and diabetes. J Fungos. (2024) 10:527. doi: 10.3390/jof10080527 39194853 PMC11355528

[16] Queiroz LAD Assis JB Guimarães JPT Sousa ESA Milhomem AC Sunahara KKS . Endangered lymphocytes: The effects of alloxan and streptozotocin on immune cells in type 1 induced diabetes. Mediators Inflammation. (2021) 2021:9940009. doi: 10.1155/2021/9940009 34712101 PMC8548114

[17] Hatch JM Segvich DM Kohler R Wallace JM . Skeletal manifestations in a streptozotocin-induced *C57BL/6* model of type 1 diabetes. Bone Rep. (2022) 17:101609. doi: 10.1016/j.bonr.2022.101609 35941910 PMC9356200

[18] Rodrigues AM De Hoog GS De Camargo ZP . Molecular diagnosis of pathogenic *Sporothrix* species. PloS NeglTrop Dis. (2015) 9:e0004190. doi: 10.1371/journal.pntd.0004190 26623643 PMC4666615

[19] Ikeda MAK Almeida JRF Jannuzzi GP Cronemberger-Andrade A Torrecilhas ACT Moretti NS . Extracellular vesicles from *Sporothrix brasiliensis* are an important virulence factor that induce an increase in fungal burden in experimental sporotrichosis. Front Microbiol Sec Microbial Phusiology Metab. (2018) 9:2286. doi: 10.3389/fmicb.2018.02286 30333803 PMC6176056

[20] Klionsky DJ Abdel-Aziz AK Abdelfatah S Abdellatif M Abdoli A Abel S . Guidelines for the use and interpretation of assays for monitoring autophagy (4th edition). Autophagy. (2021) 17:1–382. doi: 10.1080/15548627.2020.1797280 33634751 PMC7996087

[21] Zheng HJ Zhang X Guo J Zhang W Ai S Zhang F . Lysosomal dysfunction-induced autophagic stress in diabetic kidney disease. J Cell Mol Med. (2020) 24:8276–90. doi: 10.1111/jcmm.15301 32583573 PMC7412686

[22] Brazilian Diabetes Society (BDS) . Brazilian Diabetes Society Guidelines (2022). São Paulo. Available online at: https://diretriz.diabetes.org.br/ (Accessed January 13, 2026).

[23] Arrillaga-Moncrieff I Capilla J Mayayo E Marimon R Mariné R Gené J . Different virulence levels of the species of *Sporothrix* in a murine model. Clin Microbiol Infect. (2009) 15:651–5. doi: 10.1111/j.1469-0691.2009.02824.x 19624508

[24] Mario D Schaffer LF Peroza LR Jesus FPK Denardi LB Fachinetto R . *Sporothrix brasiliensis* produces the highest levels of oxidative stress in a murine model among the species of the *Sporothrix schenckii* complex. Rev Soc Bras Med Trop. (2017) 50:554–7. doi: 10.1590/0037-8682-0171-2016 28954081

[25] Almeida JRF Januzzi GP Kaihami GH Breda LCD Ferreira KS Almeida SR . An immunoproteomic approach revealing peptides from *Sporothrix brasiliensis* that induce a cellular imune response in subcutaneous sporotrichosis. Sci Rep. (2018) 8:4192. doi: 10.1038/s41598-018-22709-8 29520092 PMC5843658

[26] Kajiwara H Saito M Ohga S Uenotsuchi T Yoshida S . Impaired host defense against *Sporothrix schenckii* in mice with chronic granulomatous disease. Infect Immun. (2004) 72:5073–9. doi: 10.1128/IAI.72.9.5073-5079.2004 15322000 PMC517470

[27] Batista-Duharte A Téllez-Martínez D Roberto de Andrade C Portuondo DL Jellmayer JA Polesi MC . *Sporothrix brasiliensis* induces a more severe disease associated with sustained Th17 and regulatory T cells responses than *Sporothrix schenckii* sensu stricto in mice. Fungal Biol. (2018) 122:1163–70. doi: 10.1016/j.funbio.2018.08.004 30449354

[28] Venuti MT Roda E Brandalise F Sarkar M Cappelletti M Speciani AF . A pathophysiological intersection between metabolic biomarkers and memory: A longitudinal study in the STZ-induced diabetic mouse model. Front Physiol. (2025) 16:1455434. doi: 10.3389/fphys.2025.1455434 40144552 PMC11937145

[29] Galhardo MCG Silva MTT Lima MA Nunes EP Schettini LEC De Freitas RF . *Sporothrix schenckii* meningitis in AIDS during imune reconstitution syndrome. J Neurol Neurosurg Psychiatry. (2010) 81:696–9. doi: 10.1136/jnnp.2009.173187 20392979

[30] Silva-Vergara ML De Camargo ZP Silva PF Abdalla MR Sgarbieri RN Rodrigues AM . Case report: Disseminated *Sporothrix brasiliensis* infection with endocardial and ocular involvement in an HIV-infected patient. Am J Trop Med Hyg. (2012) 86:477–80. doi: 10.4269/ajtmh.2012.11-0441 22403321 PMC3284366

[31] Freitas DFS Lima MA Almeida-Paes R Lamas CC Do Valle ACF Oliveira MME . Sporotrichosis in the central nervous system caused by *Sporothrix brasiliensis*. Clin Infect Dis. (2015) 15:663–4. doi: 10.1093/cid/civ361 25956895

[32] Freitas DFS Santos SS Almeida-Paes R De Oliveira ME Do Valle ACF Gutierrez-Galhardo MC . Increase in virulence of *Sporothrix brasiliensis* over five years in a patient with chronic disseminated sporotrichosis. Virulence. (2015) 6:112–20. doi: 10.1080/21505594.2015.1014274 25668479 PMC4601271

[33] Moreira JAS Freitas DFS Lamas CC . The impact of sporotrichosis in HIV-infected patients: A systematic review. Infection. (2015) 43:267–76. doi: 10.1007/s15010-015-0746-1 25701221

[34] Xia X Zhi H Liu Z . Cutaneous disseminated sporotrichosis associated with diabetes: A case report and literature review. PloS Negl Trop Dis. (2023) 17:e0011647. doi: 10.1371/journal.pntd.0011647 37721953 PMC10538719

[35] Rossato L Dos Santos SS Ferreira LG De Almeida SR . The impact of the absence of Toll-like receptor-2 during *Sporothrix brasiliensis* infection. J Med Microbiol. (2019) 68:87–94. doi: 10.1099/jmm.0.000876 30451650

[36] Della Terra PP Rodrigues AM Fernandes GF Nishikaku AS Burger E De Camargo ZP . Exploring virulence and immunogenicity in the emerging pathogen *Sporothrix brasiliensis*. PloS NeglTrop Dis. (2017) 11:e0005903. doi: 10.1371/journal.pntd.0005903 28854184 PMC5595342

[37] Manente FA Quinello C Ferreira LS De Andrade CR Jellmayer JA Portuondo DL . Experimental sporotrichosis in a cyclophosphamide-induced immunosuppressed mice model. Med Mycol. (2018) 56:711–22. doi: 10.1093/mmy/myx098 29087533

[38] Nakamizo S Kabashima K . Cutaneous granulomas: Mechanisms, cellular interactions and therapeutic insights. Br J Dermatol. (2025) 192:974–82. doi: 10.1093/bjd/ljaf096 40080709

[39] Borges BM Ramos RBC Preite NW Kaminski VL Alves de Castro P Camacho M . Transcriptional profiling of a fungal granuloma reveals a low metabolic activity of *Paracoccidioides brasiliensis* yeasts and an actively regulated host immune response. Front Cell Infect Microbiol. (2023) 13:1268959. doi: 10.3389/fcimb.2023.1268959 37868350 PMC10585178

[40] Jalkanen S Salmi M . Lymphocyte adhesion and trafficking. In: Clinical Immunology, 1st ed, vol. 11. Elsevier, Amsterdam, The Netherlands (2019). p. 171–82. doi: 10.1016/B978-0-7020-8165-1.00016-2

[41] Van de Veerdonk FL Netea MG . T-cell subsets and antifungal host defenses. Curr Fungal Infect Rep. (2010) 4:238–43. doi: 10.1007/s12281-010-0034-6 20976286 PMC2949562

[42] Batista-Duharte A Téllez-Martínez D Portuondo DL Carlos IZ . Selective depletion of regulatory T cells enhances the immunogenicity of a recombinant-based vaccine against *Sporothrix spp*. Front Cell Infect Microbiol. (2023) 10:12:1084526. doi: 10.3389/fcimb.2022.1084526 36846549 PMC9951613

[43] Yagnik KJ Skelton W Olson A Trillo CA Lascano J . A rare case of disseminated *Sporothrix schenckii* with bone marrow involvement in a patient with idiopathic CD4 lymphocytopenia. IDCases. (2017) 9:70–2. doi: 10.1016/j.idcr.2017.06.012 28706855 PMC5503829

[44] Magalhães VCR Colombo SA Freitas GJC Moura AS Vieira FCL Lyon AC . Late diagnosis of disseminated *Sporothrix brasiliensis* infection with bone marrow involvement in an HIV-negative patient. Pathogens. (2022) 11:1516. doi: 10.3390/pathogens11121516 36558850 PMC9781367

[45] Sassá MF Ferreira LS Ribeiro LC Carlos IZ . Immune response against *Sporothrix schenckii* in TLR-4-deficient mice. Mycopathologia. (2012) 174:21–30. doi: 10.1007/s11046-012-9523-1 22286932

[46] Grosick R Alvarado-Vazquez PA Messersmith AR Romero-Sandoval EA . High glucose induces a priming effect in macrophages and exacerbates the production of pro-inflammatory cytokines after a challenge. J Pain Res. (2018) 11:1769–78. doi: 10.2147/JPR.S164493 30237731 PMC6136416

[47] Galvão Tessaro FH Ayala TS Bella LM Martins JO . Macrophages from a type 1 diabetes mouse model present dysregulated Pl3K/AKT, ERK 1/2 and SAPK/JNK levels. Immunobiology. (2020) 225:151879. doi: 10.1016/j.imbio.2019.11.014 31812346

[48] Tseng CH Shah KM Chiu IJ Hsiao LL . The role of autophagy in type 2 diabetic kidney disease management. Cells. (2023) 12:2691. doi: 10.3390/cells12232691 38067119 PMC10705810

[49] Kanayama M Shinohara ML . Roles of autophagy and autophagy-related proteins in antifungal immunity. Front Immunol. (2016) 7:47. doi: 10.3389/fimmu.2016.00047 26925060 PMC4757664

[50] Lamark T Kirkin V Dikic I Johansen T . NBR1 and p62 as cargo receptors for selective autophagy of ubiquitinated targets. Cell Cycle. (2009) 8:1986–90. doi: 10.4161/cc.8.13.8892 19502794

[51] Sunahara KK Nunes FP Baptista MA Strell C Sannomiya P Westerberg LS . Insulin influences autophagy response distinctively in macrophages of different compartments. Cell Physiol Biochem. (2014) 34:2017–26. doi: 10.1159/000366397 25562150

[52] Abe H Uchida T Hara A Mizukami H Komiya K Koike M . Exendin-4 improves β-cell function in autophagy-deficient β-cells. Endocrinology. (2013) 154:4512–24. doi: 10.1210/en.2013-1578 24105478

[53] Hernández-Chávez M Pérez-García LA Ninõ-Veja GA Mora-Montes HM . Fungal strategies to evade the host immune recognition. J Fungi (Basel). (2017) 3:51. doi: 10.3390/jof3040051 29371567 PMC5753153

[54] Guan M Yao L Zhen Y Song Y Liu X Liu Y . *Sporothrix globosa* melanin regulates autophagy via the TLR2 signaling pathway in THP-1 macrophages. PloS NeglTrop Dis. (2023) 17:e0011281. doi: 10.1371/journal.pntd.0011281 37141335 PMC10198546

[55] Wang JE Muralidharan C Nargis T Enriquez JR Anderson RM Mirmira RG . The integrated stress response promotes macrophage inflammation and migration in autoimmune diabetes. Cell Commun Signaling. (2025) 23:368. doi: 10.1186/s12964-025-02372-z 40836297 PMC12366006

