# Supplementary material for: Diabetes impairs antifungal immunity and drives fatal disseminated sporotrichosis in mice
Source: Front Immunol. 2026 Sep 3;17:1902740. doi: 10.3389/fimmu.2026.1902740 (PMC13581713; doi:10.3389/fimmu.2026.1902740)
Supplement: Supplementary file 1 [file DataSheet1.pdf]

frontiers

The International Society for the Advancement of Cytometry (ISAC) has highlighted the importance of including comprehensive methodological information to ensure data reproducibility and reliability. In line with this, Frontiers in Immunology now requires authors to submit a checklist for manuscripts that involve flow or mass cytometry. This checklist helps standardize the reporting process, improving the quality and transparency of published data. By doing so, we support scientific progress, making it easier for other researchers to replicate and validate experiments.

This form should be submitted with any manuscripts using flow or mass cytometry.

Sample/specimen/material description

☐ Total blood

☐ PBMCs

☐ Organ digests

Other Lymph node, spleen, and tissue lesion

Did the samples suffer any treatment before or after incubation with the antibodies?

☐ Drug ___________________________________

☐ Cell permeabilization ______________________

☐ Dye ____________________________________

☐ Propidium iodine

☐ Not applicable

Other CFSE (Carboxifluoresceína Succinimidil Éster) - phagocytosis assays

Instrument and antibodies

Name of the Cytometer FACS CANTO II (BD Bioscences, San Jose, CA, USA)

Antibodies and targets	Fluorochrome/ Metal	Catalog number/Company
e.g. anti-CD4	FITC	Cat. XXX/ XXX ltd.
CD3	APC	565643/ BD biosciences

CD4	FITC	553729/ BD biosciences
CD8a	PE	553033/ BD biosciences
CD45R	APC-Cy7	552094/ BD biosciences
CD19	PE-Cy7	552854/ BD biosciences
IgM	PerCP-Cy5.5	550881/ BD biosciences
CD11c	APC-Cy7	561241/ BD biosciences
CD11b	PE-Cy7	552850/ BD biosciences
GR1	APC	553129/ BD biosciences
F4/80	PE	565410/ BD biosciences
MHCII	FITC	553551/ BD biosciences

Data analyses

1. Name of the software FlowJo (Orlando, FL, USA)______
2. Reference gating strategy in the manuscript or supplementary material

Gating strategy in (eg Figure X) The gating strategy can be found in the supplementary materials
