# Supplementary material for: Diabetes impairs antifungal immunity and drives fatal disseminated sporotrichosis in mice
Source: Front Immunol. 2026 Sep 3;17:1902740. doi: 10.3389/fimmu.2026.1902740 (PMC13581713; doi:10.3389/fimmu.2026.1902740)
Supplement: Supplementary file 2 [file DataSheet2.pdf]

**ELECTRONIC SUPPLEMENTARY MATERIAL (ESM)**

**ESM Fig. 1:** Photos of PCR gel confirming the *Sporothrix brasiliensis* strain:

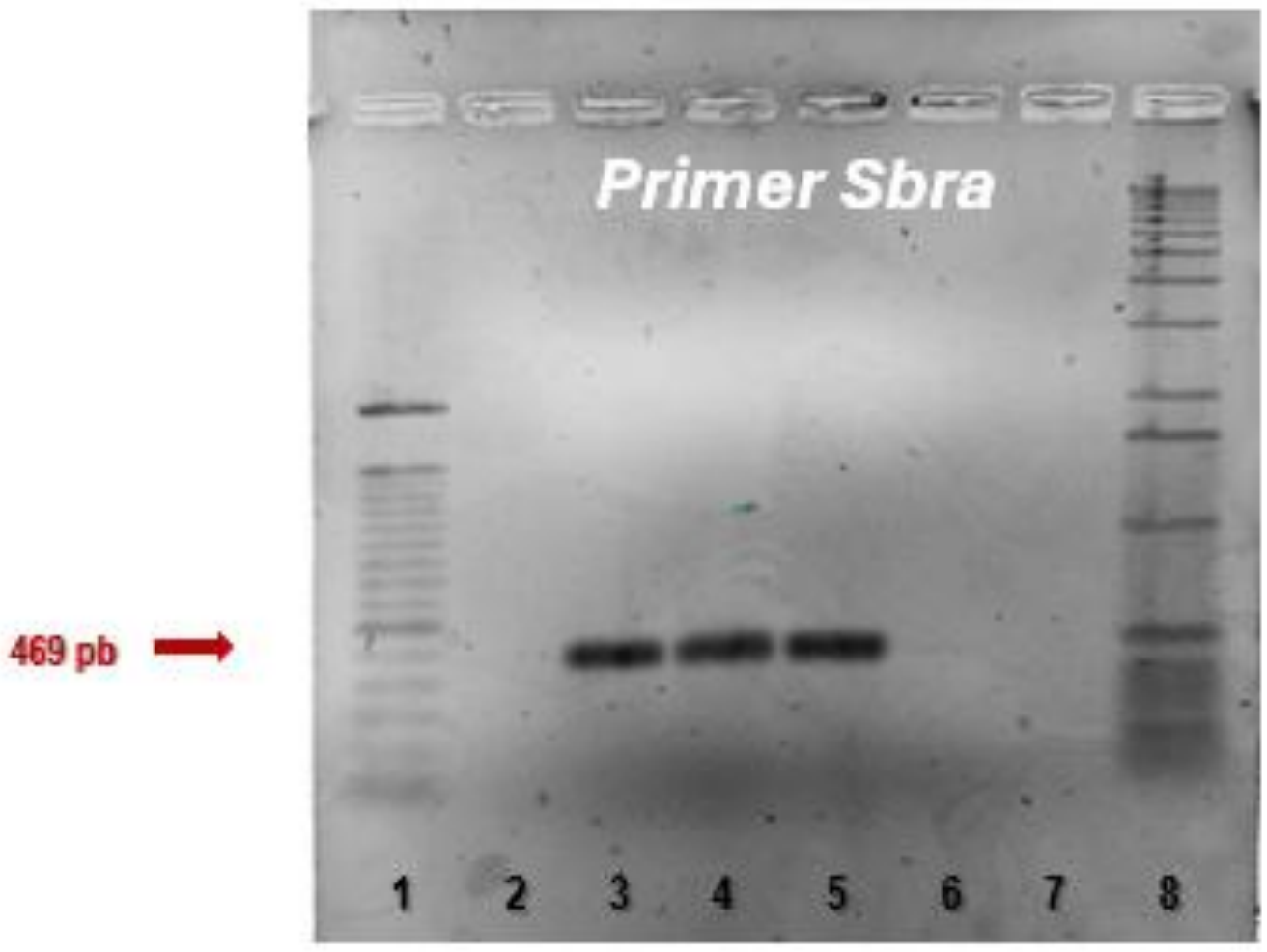


Lysis of strains of *Sporothrix schenckii*, *Sporothrix brasiliensis*, and *Candida albicans*. Electrophoretic separation was performed on 1% agarose gel for 120 minutes. *Sporothrix brasiliensis* (469 bp gene) **1** - 100 bp molecular weight standard, **2** - *Sporothrix schenckii*, **3** - *Sporothrix brasiliensis* M1168, **4** - *Sporothrix brasiliensis* M1168, **5** - *Sporothrix brasiliensis* 5110, **6** - *Candida albicans*, **7** - Negative control.

**ESM Fig. 2: photos of western blotting membranes**:

In all membranes, the groups in the gel are in the following order in the bands:

1. Control
2. Control treated with rapamycin (result not used in this article)
3. Diabetic
4. Diabetic treated with rapamycin (result not used in this article)
5. Control + *S. brasiliensis* M1168
6. Control + *S. brasiliensis* M1168 treated with rapamycin (result not used in this article)
7. Diabetic + *S. brasiliensis* M1168
8. Diabetic + *S. brasiliensis* M1168 treated with rapamycin (result not used in this article)

Beclin-1 and β-*actin* (12% SDS-PAGE gel):

N1

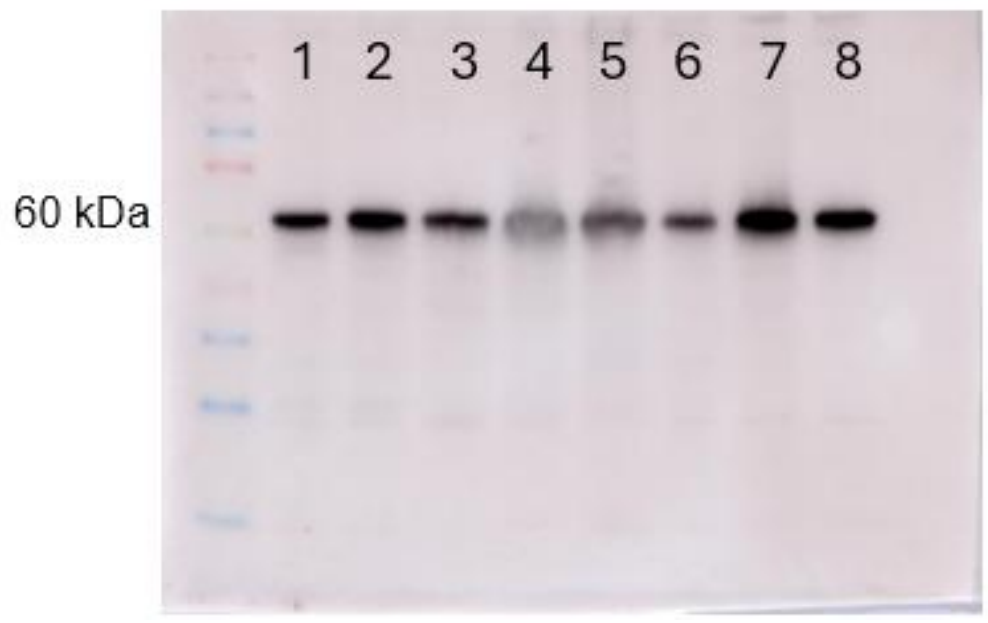


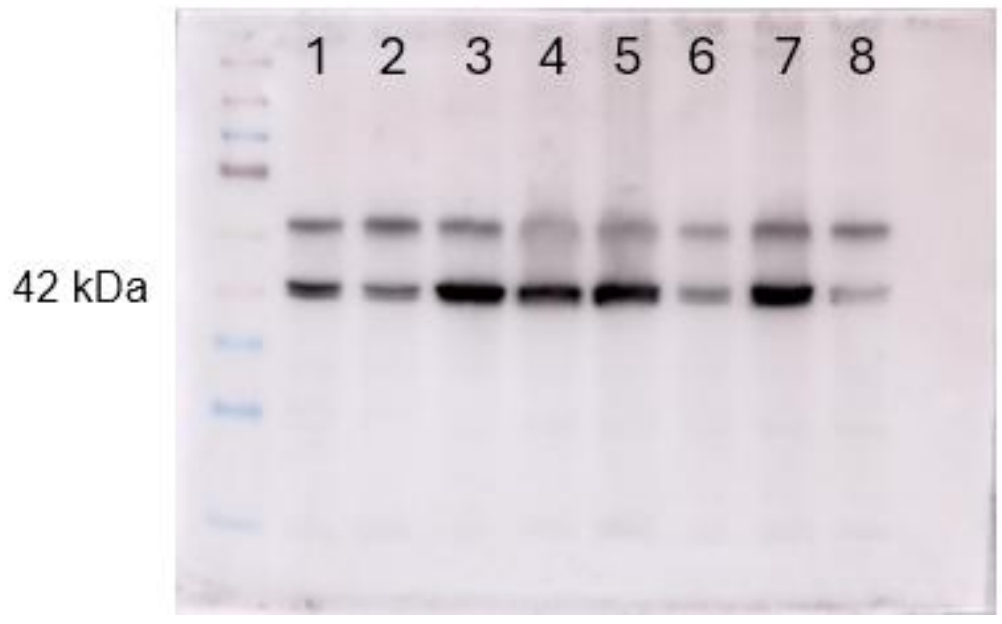


N2

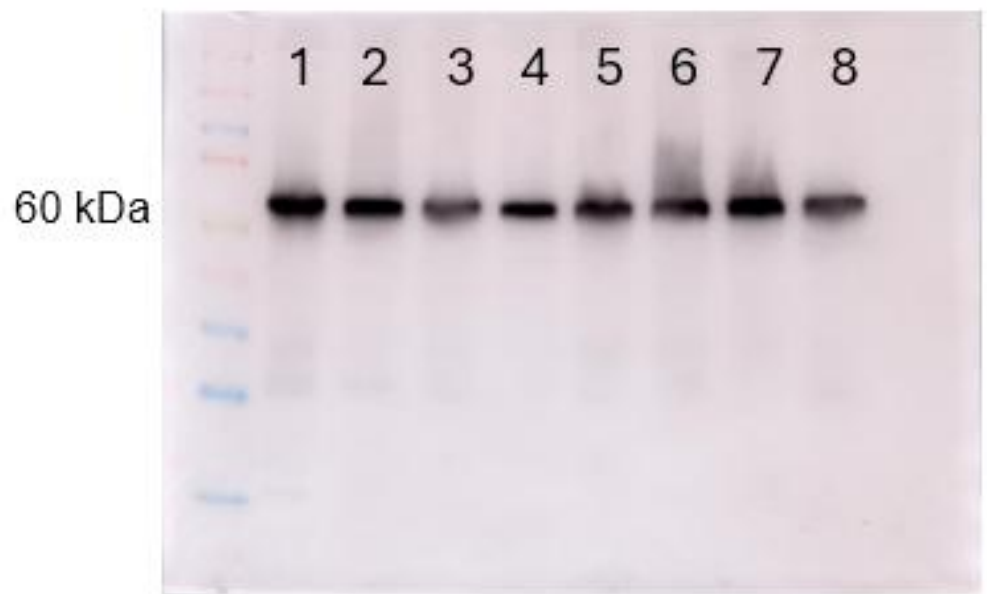


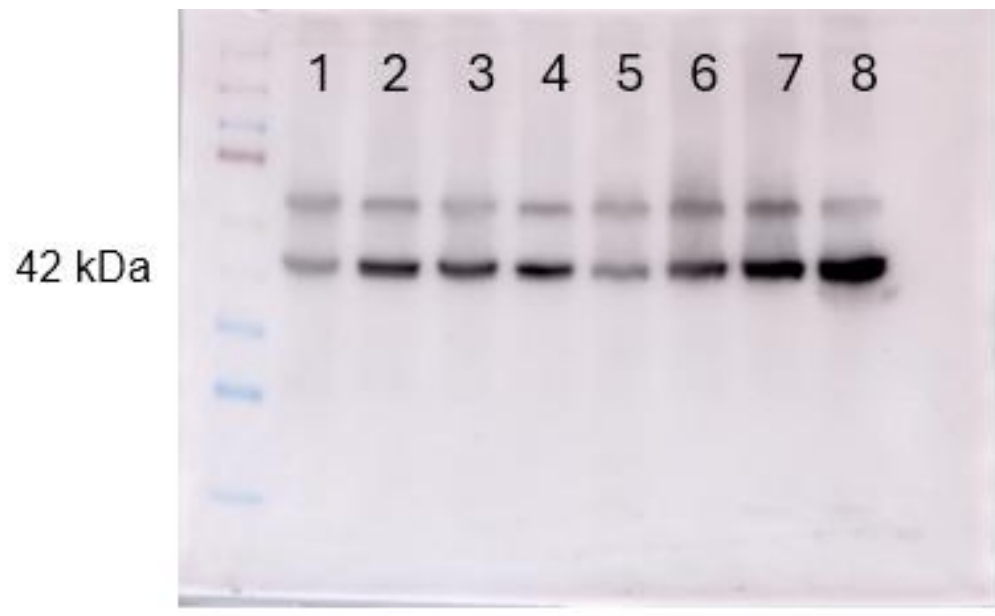


N3

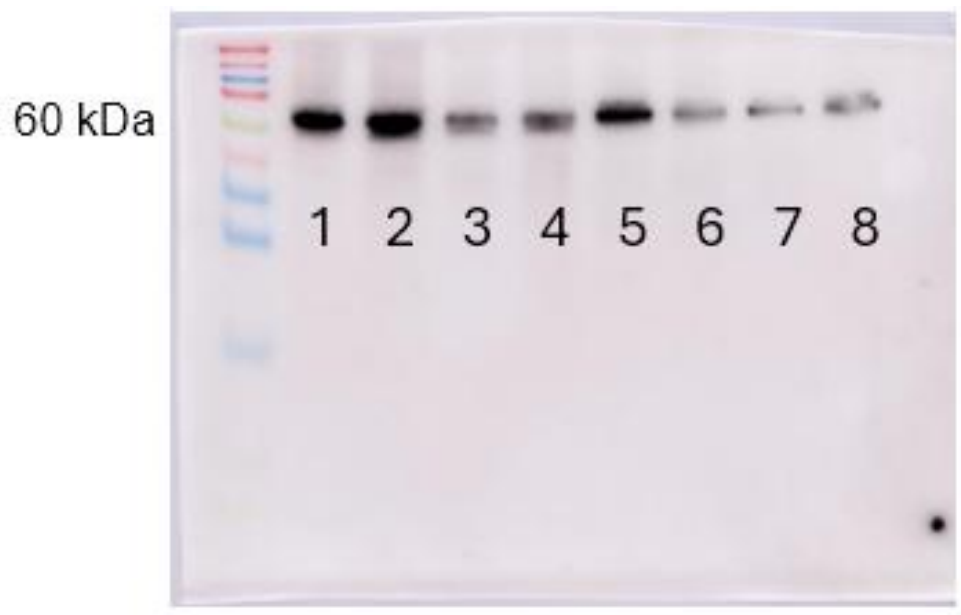


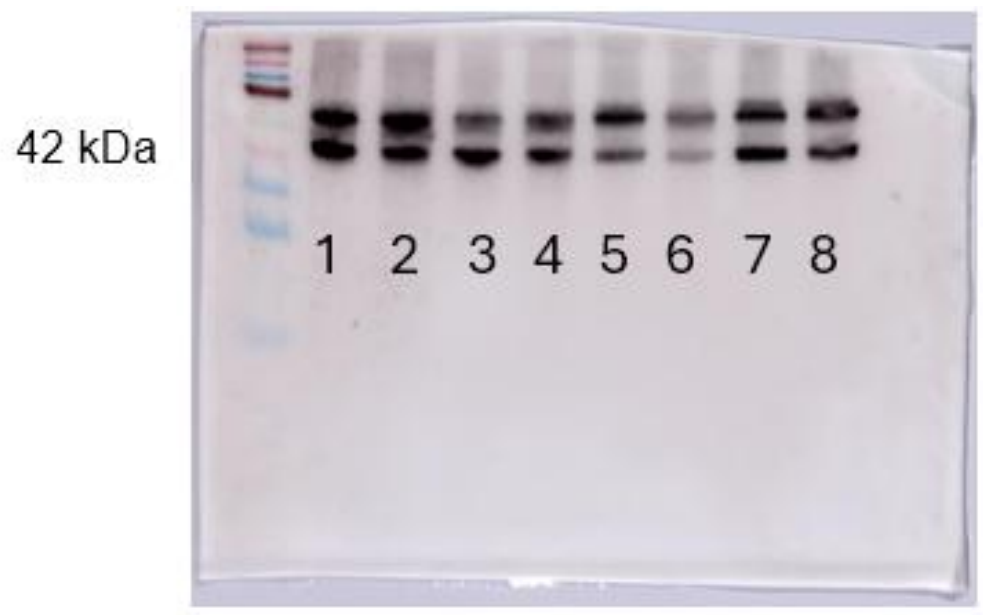


N4

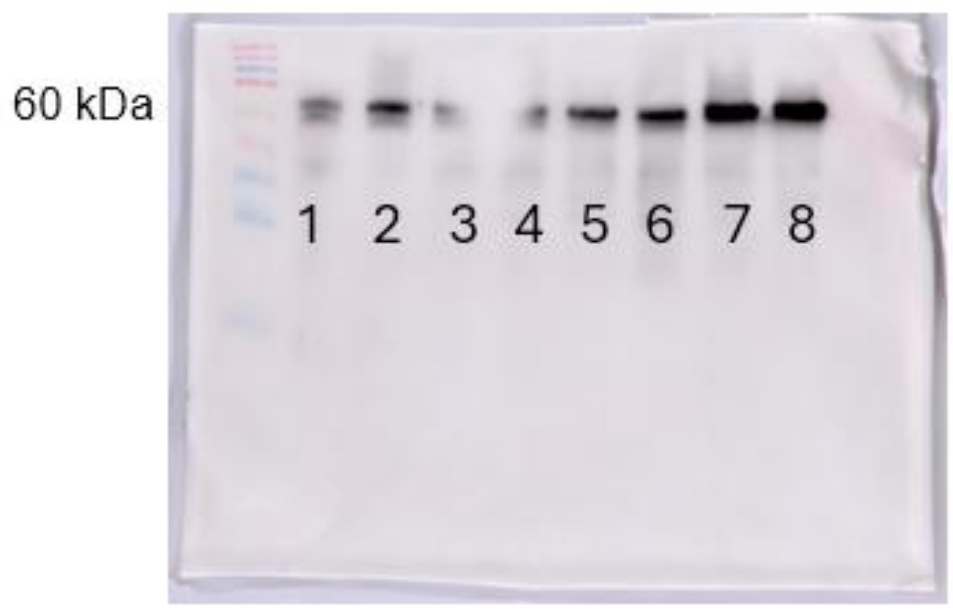


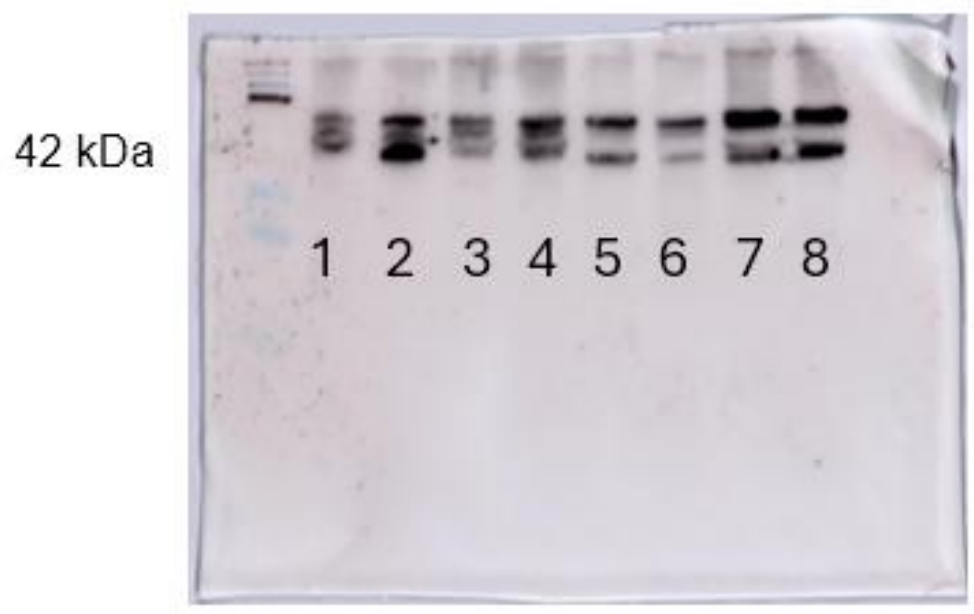

N5

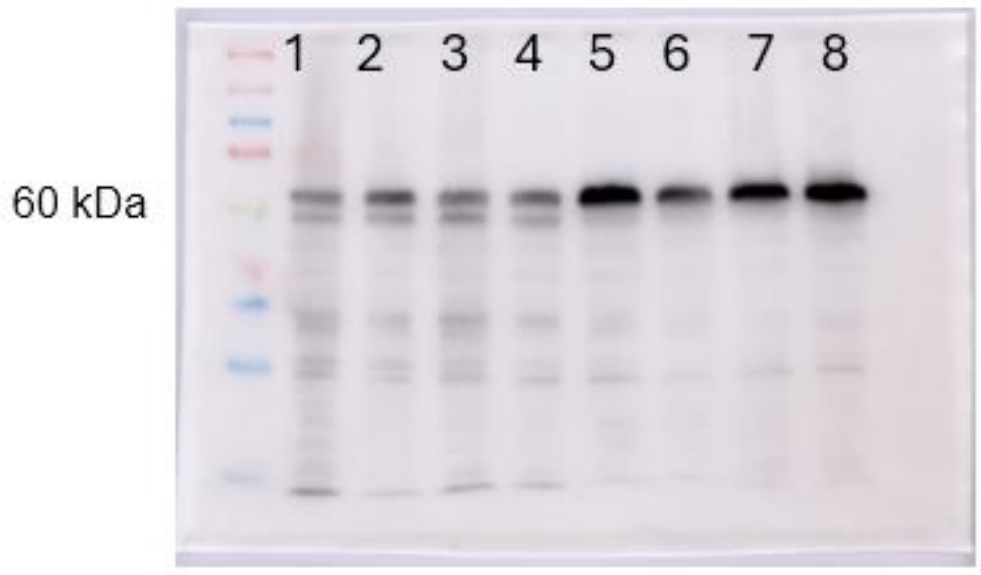


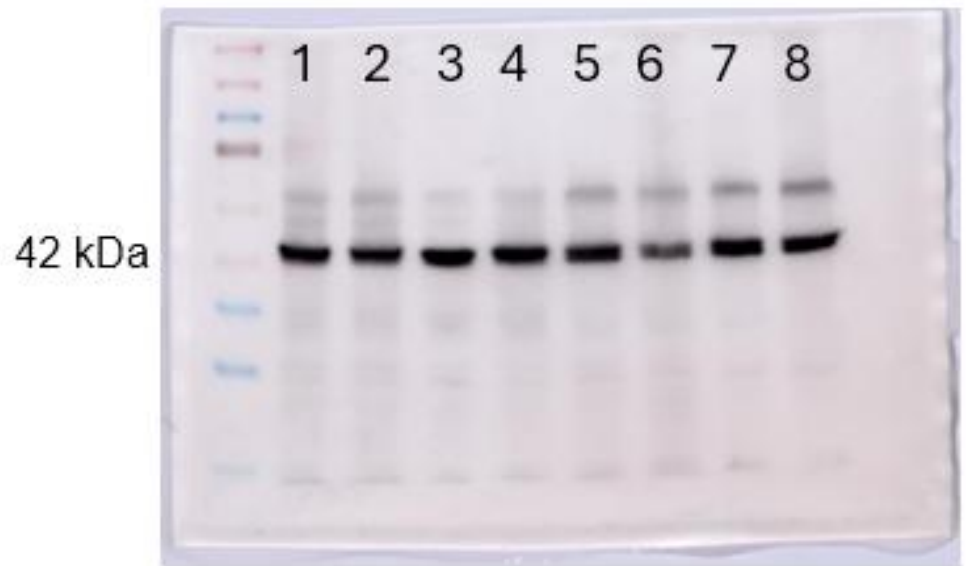


N6

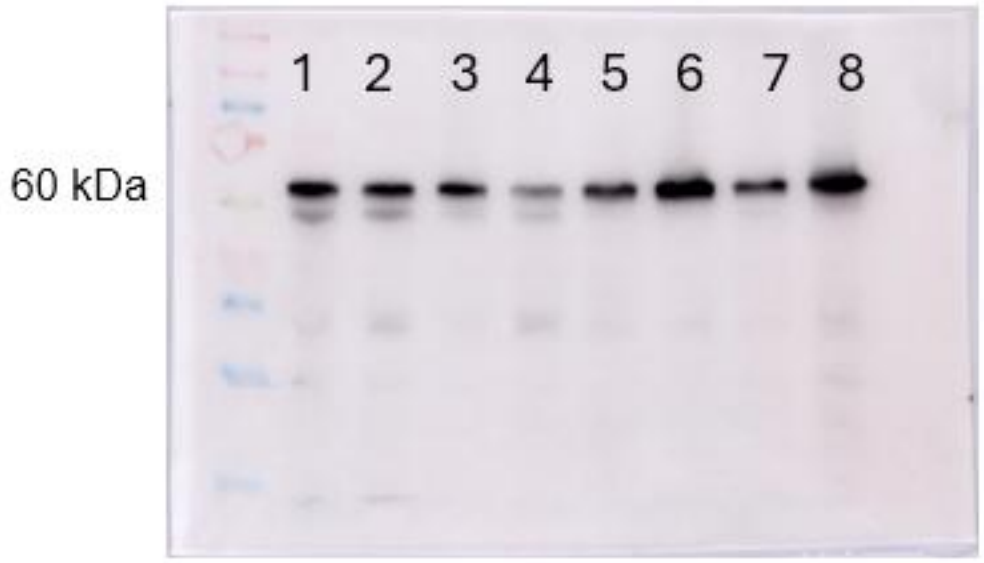


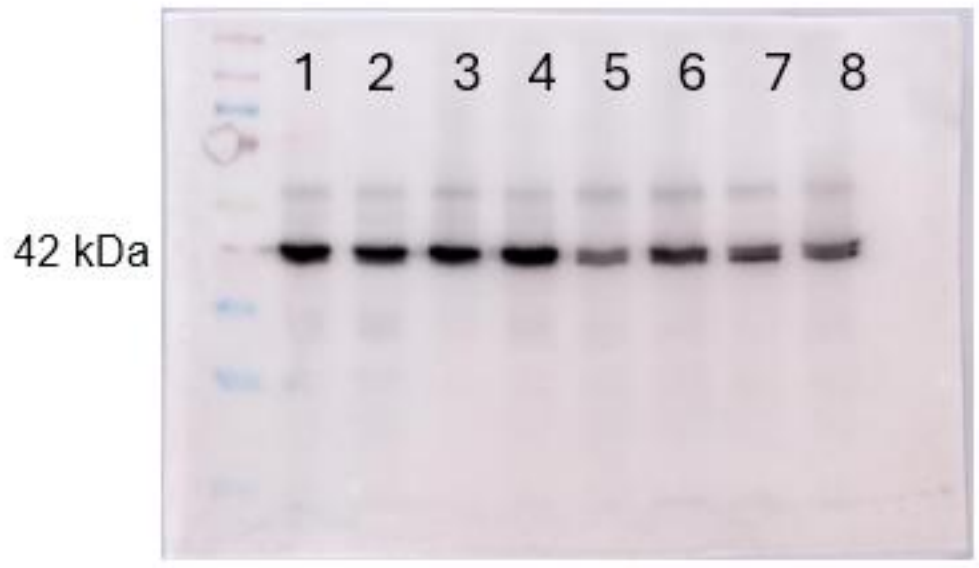


LC3B-II and β-*actin* (12% SDS-PAGE gel):

N1

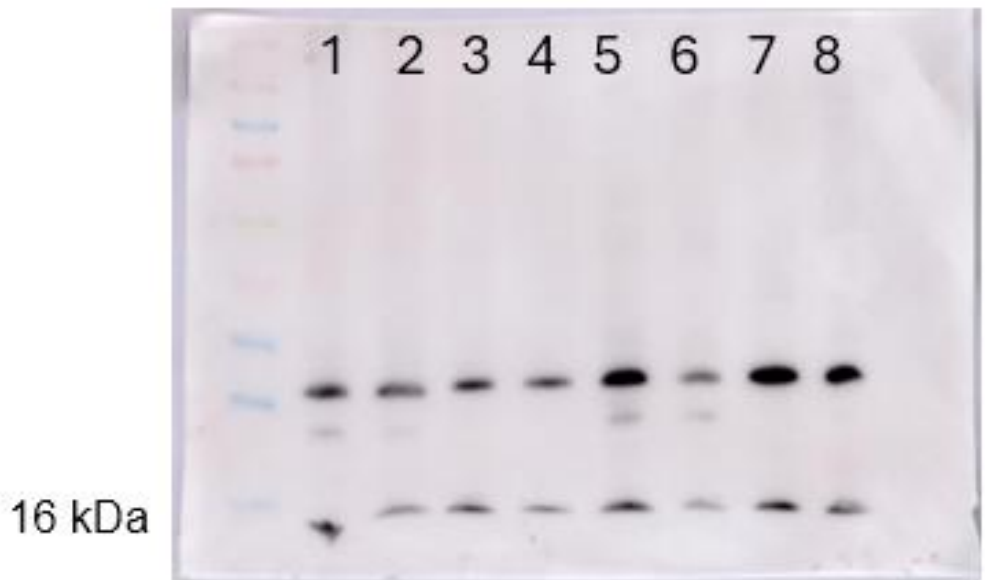


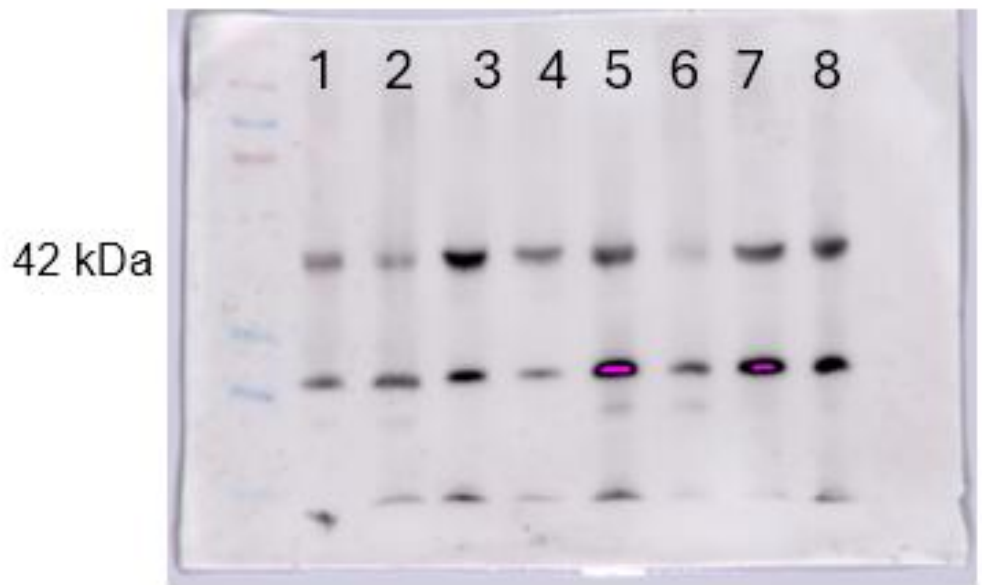


N2

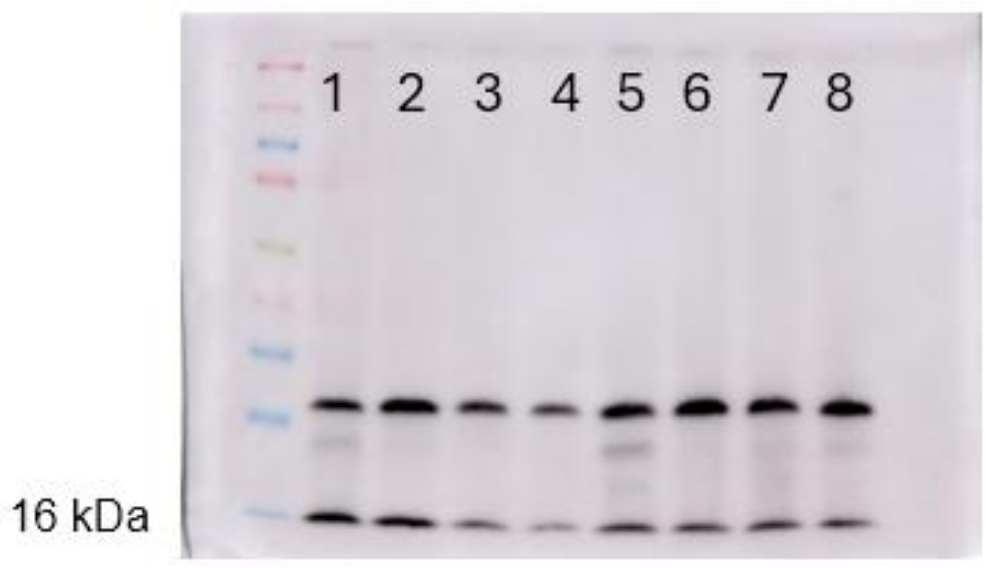


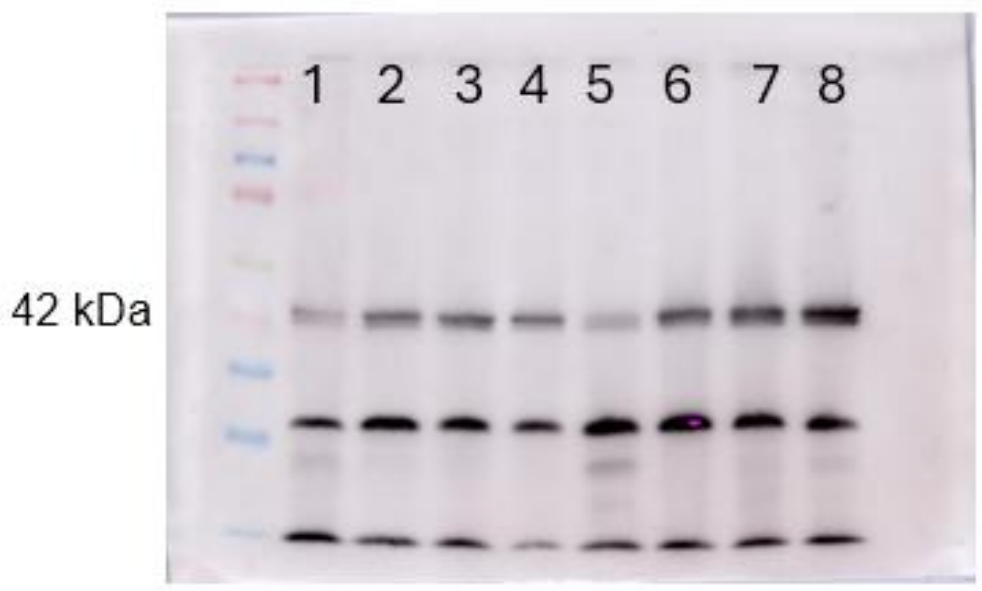

N3

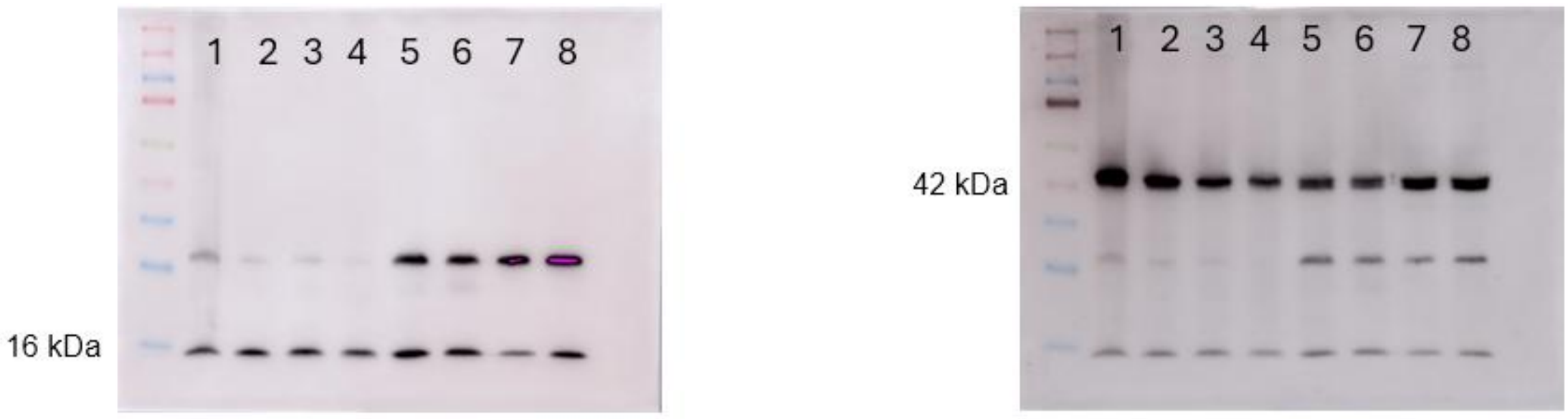


N4

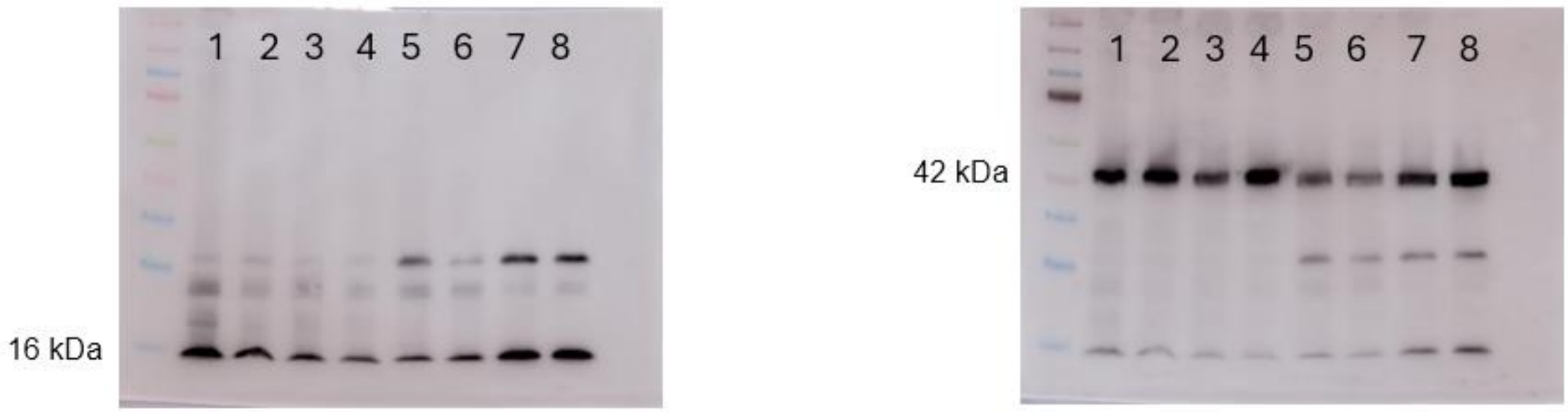


N5

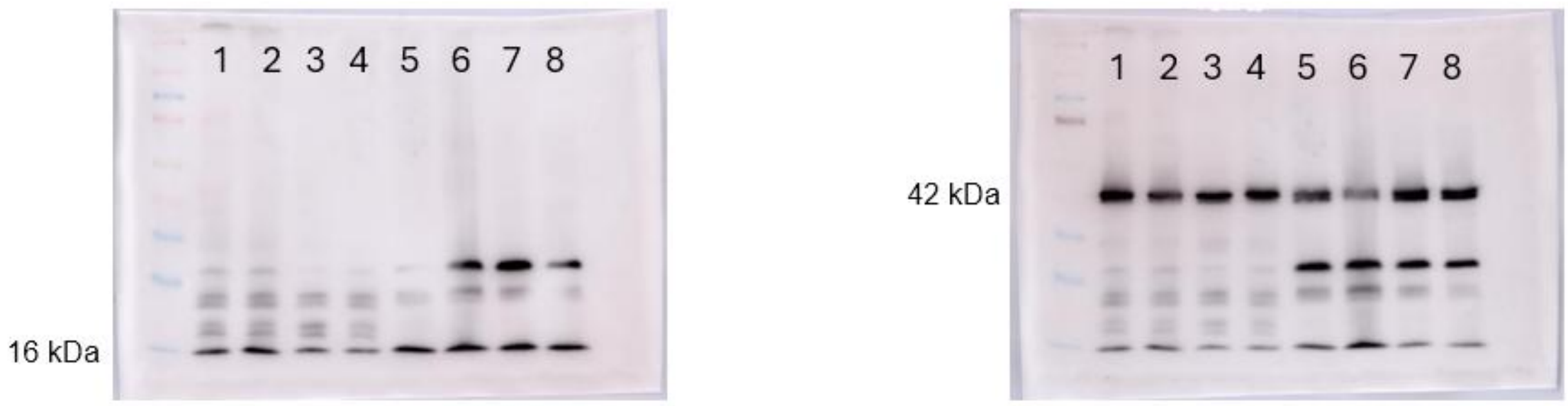


N6

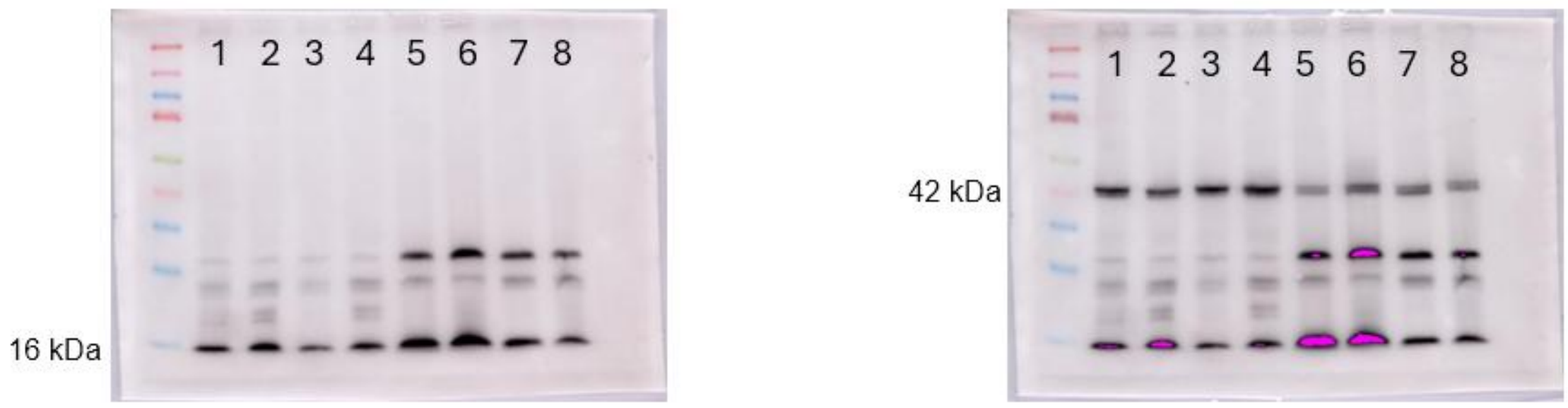


p62 and β-*actin* (12% SDS-PAGE gel):

N1

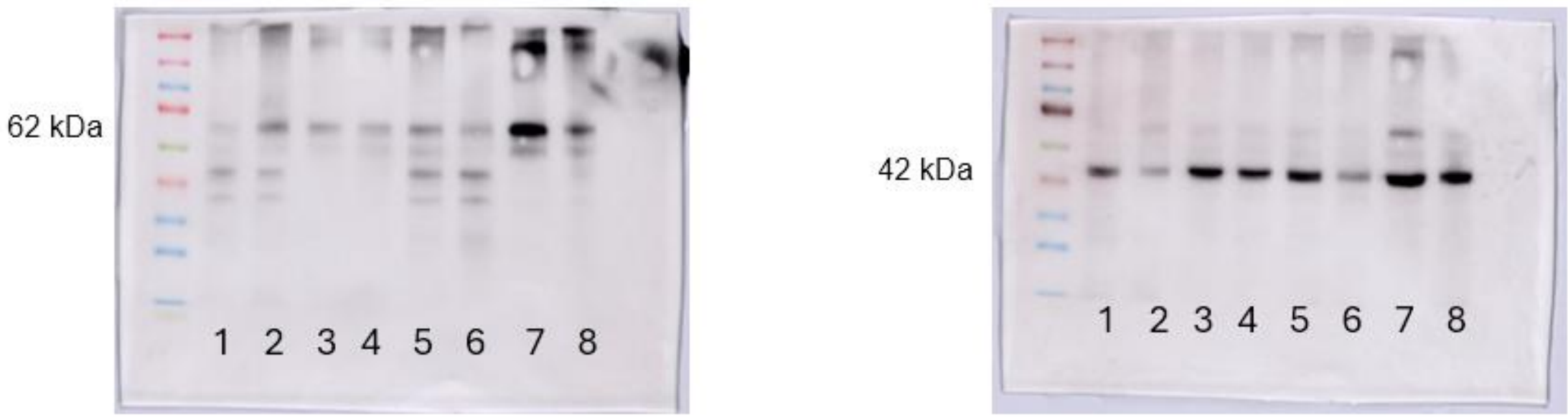
62 kDa
1 2 3 4 5 6 7 8
42 kDa
1 2 3 4 5 6 7 8

N2

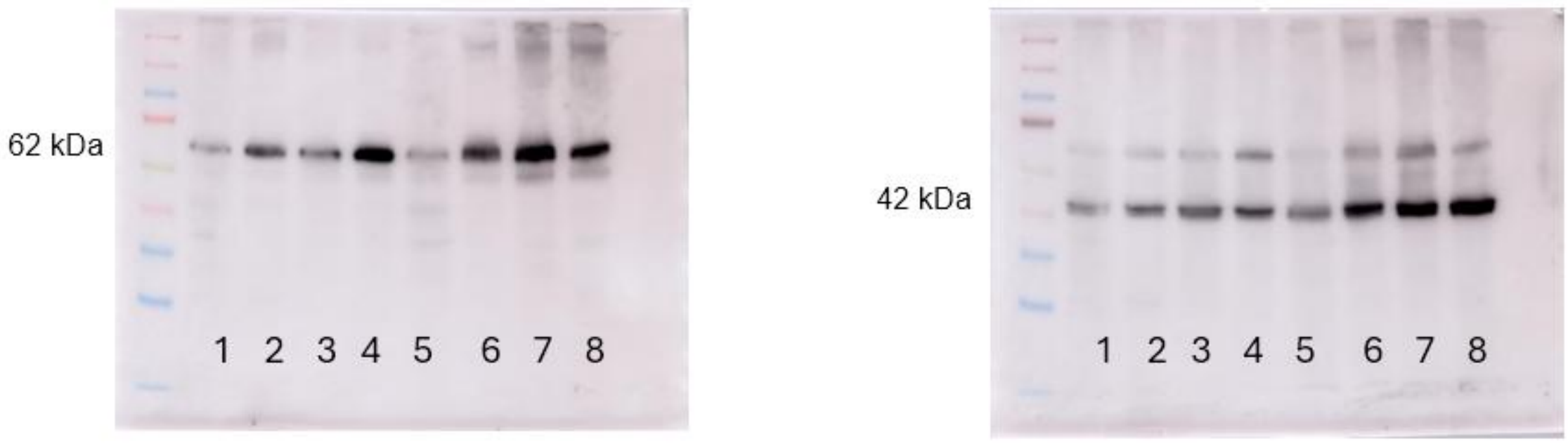
62 kDa
1 2 3 4 5 6 7 8
42 kDa
1 2 3 4 5 6 7 8

N3

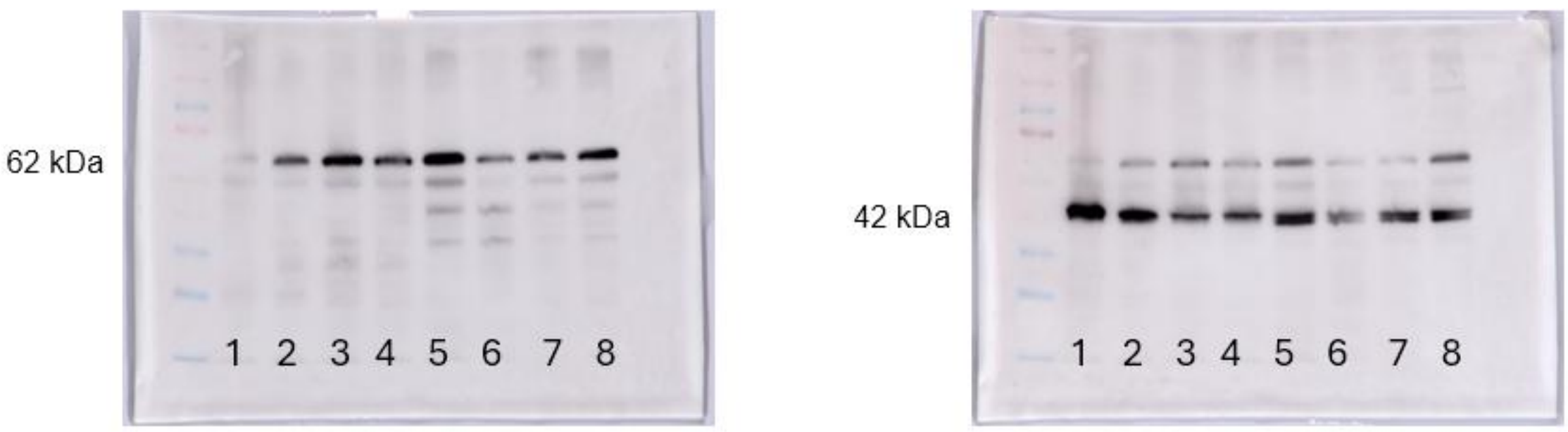
62 kDa
1 2 3 4 5 6 7 8
42 kDa
1 2 3 4 5 6 7 8

N4

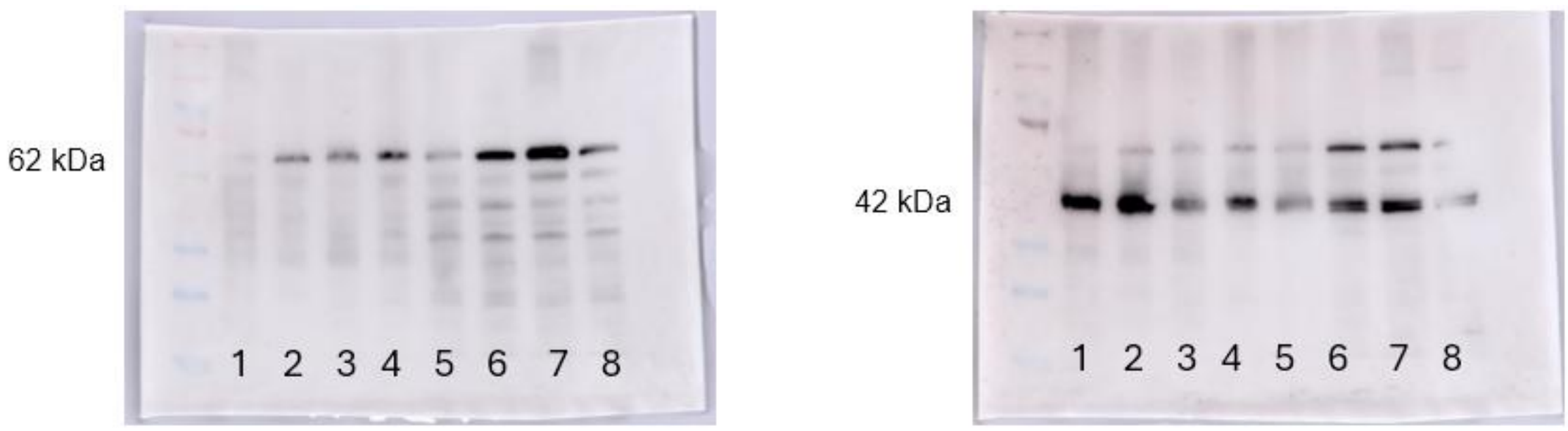
62 kDa
1 2 3 4 5 6 7 8
42 kDa
1 2 3 4 5 6 7 8

N5

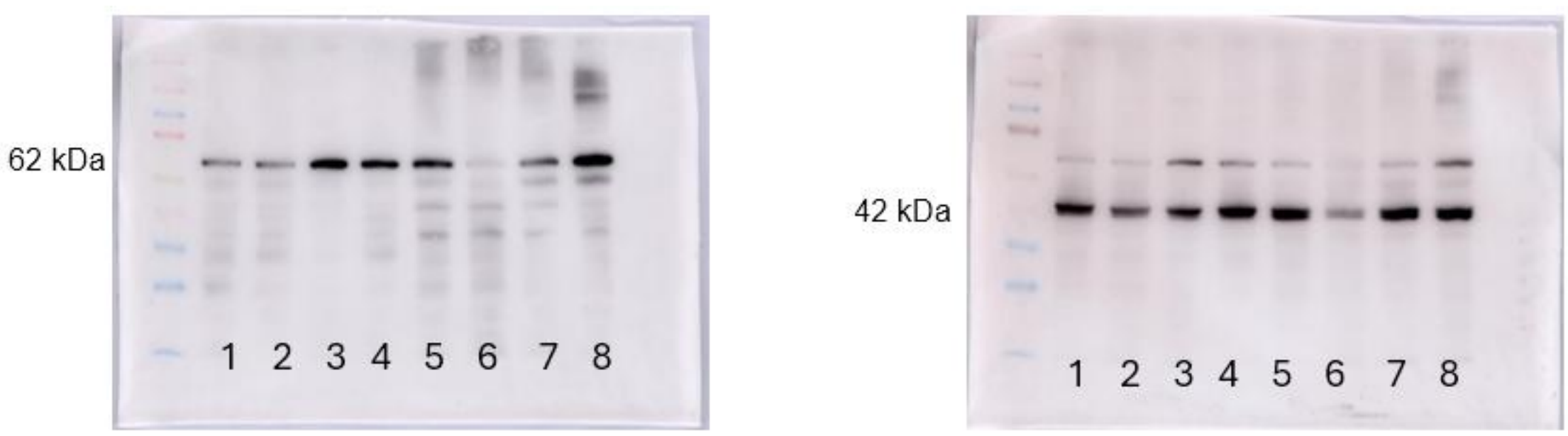
62 kDa
1 2 3 4 5 6 7 8
42 kDa
1 2 3 4 5 6 7 8

N6

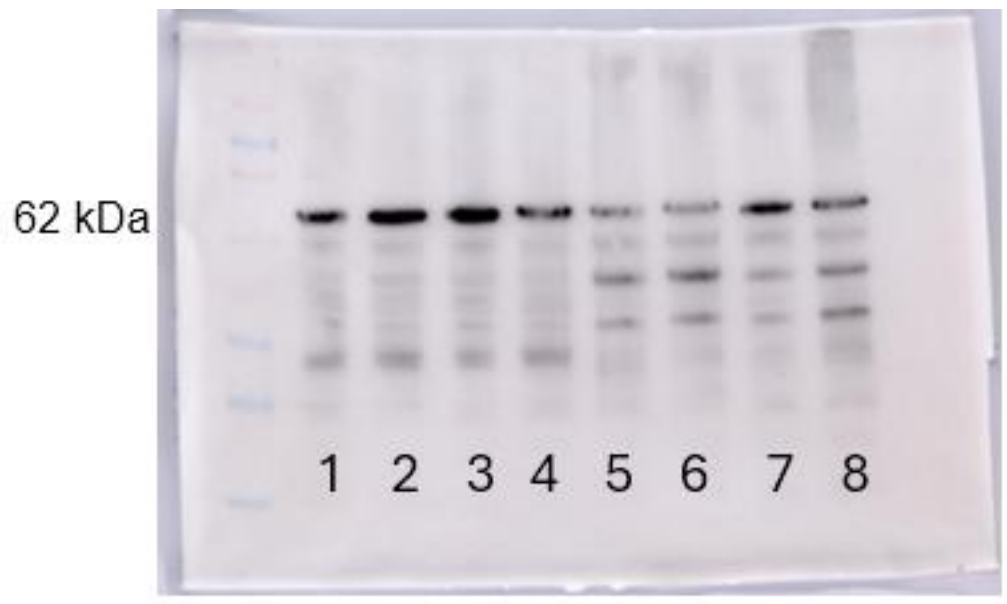


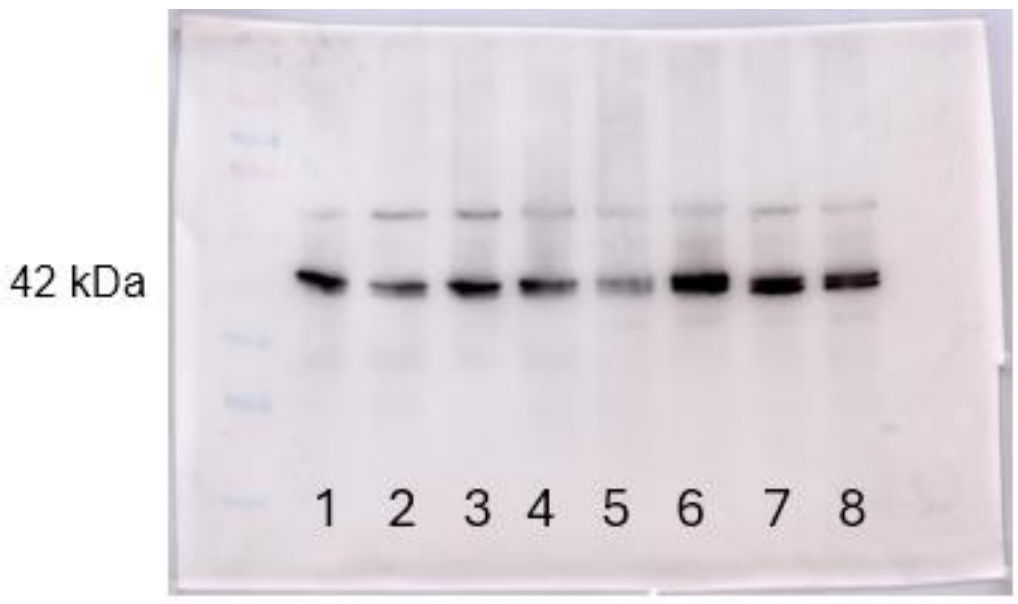


**ESM Table 1:**

**Immunophenotyping and flow cytometry:** Monoconal antibodies used.

| Antibody | Fluorochrome | Clone | Brand | CAT. NO |
|---|---|---|---|---|
| **CD3** | APC | 17A2 | BD biosciences | 565643 |
| **CD4** | FITC | GK1.5 | BD biosciences | 553729 |
| **CD8a** | PE | 53-6.7 | BD biosciences | 553033 |
| **CD45R** | APC-Cy7 | RA3-6B2 | BD biosciences | 552094 |
| **CD19** | PE-Cy7 | 1D3 | BD biosciences | 552854 |
| **IgM** | PerCP-Cy5.5 | R6-60.2 | BD biosciences | 550881 |
| **CD11c** | APC-Cy7 | HL3 | BD biosciences | 561241 |
| **CD11b** | PE-Cy7 | M1/70 | BD biosciences | 552850 |
| **GR1** | APC | RB6-8C5 | BD biosciences | 553129 |
| **F4/80** | PE | T45-2342 | BD biosciences | 565410 |
| **MHCII** | FITC | AF6-120.1 | BD biosciences | 553551 |

**ESM Table 2:**

**Molecular Identification by PCR**: Species-specific primer sequences targeting the calmodulin (*CAL*) gene in members of the genus *Sporothrix* (Rodrigues *et al*., 2015*)*.

| Target Species | Primer | Primer Sequence (5´-3`) | Target | Amplicon Size (bp) |
|---|---|---|---|---|
| ***S. brasiliensis*** | *Sbra-F* | CCC CCG TTT GAC GCT TGG | *CAL* | 469 |
| | *Sbra-R* | CCC GGA TAA CCG TGT GTC ATA AT | | |
| ***S. schenckii*** | *Ssch-F* | TTT CGA ATG CGT TCG GCT GG | *CAL* | 331 |
| | *Ssch-R* | CTC CAG ATC ACC GTG TCA | | |
| **Internal positive control (*IPC*)** | *IPC-F* | ATG CGA TAC GTA ATG TGA ATT GC | 5.8S | 101 |
| | *IPC-R* | GAC GCT CGG ACA GGC ATG | | |

**ESM Fig. 3: Gates used in flow cytometry:**

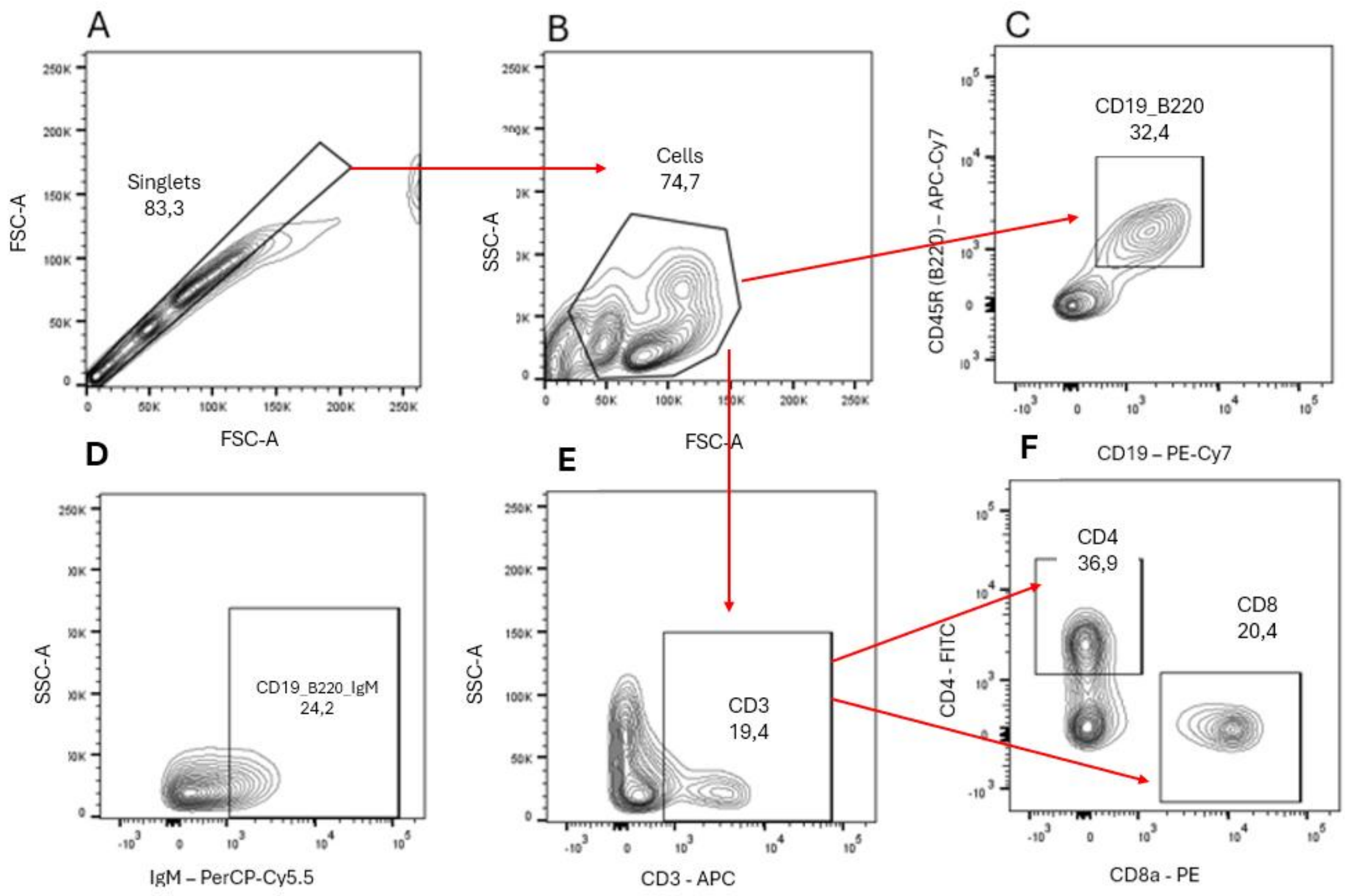

**Gating strategy for flow cytometric immunophenotyping of lymphocyte subsets. Representative flow cytometry gating strategy utilized for leukocyte analysis in lymphoid tissues (corresponding to Figure 7).** Red arrows indicate the sequential gating hierarchy**.** (**A**) Discrimination and exclusion of cell doublets to isolate single cells (singlets, *FSC-H* vs. *FSC-A*). (**B**) Morphometric gating on intact leukocyte populations (*FSC-A* vs. *SSC-A*). (**C–F**) Identification and subset characterization of lymphocyte populations using specific fluorophore-conjugated antibodies (as listed in ESM Table 1): (**C**) Total B cells (CD19+CD45R/B220), (**D**) Mature B cell subset (IgM+), (**E**) Total T lymphocytes (CD3+), and (**F**) Distinct T cell subsets expressing CD4+ (T helper) or CD8a+ (cytotoxic T cells) gated within the CD3+ population.

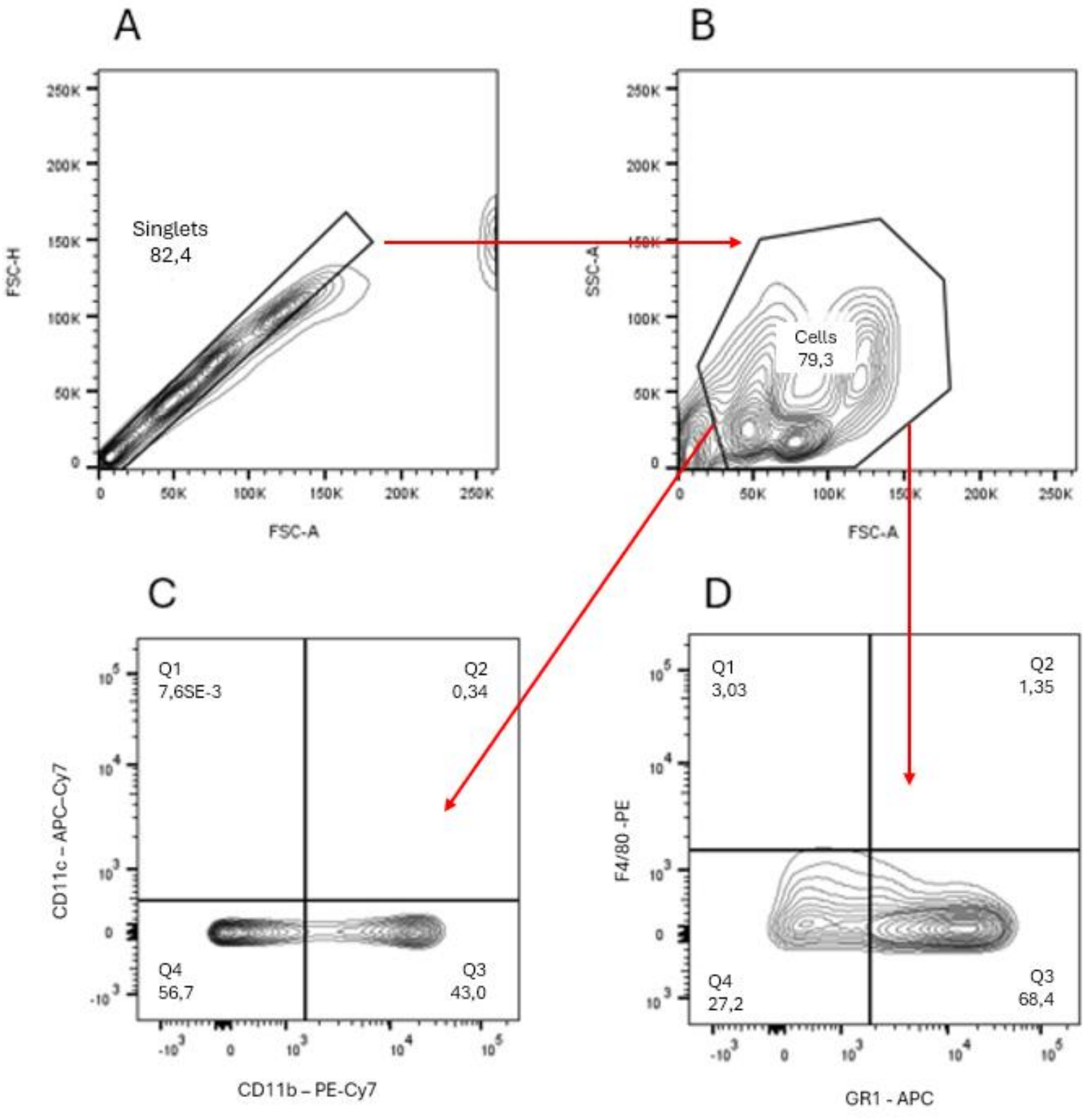

**Gating strategy for flow cytometric immunophenotyping of myeloid cell populations in skin lesions.** Representative flow cytometry gating strategy utilized for myeloid lineage characterization in cutaneous tissue (corresponding to Figure 8). Red arrows indicate the sequential gating hierarchy. (**A**) Exclusion of cell doublets to isolate single cells (singlets, *FSC-H* vs. *FSC-A*). (**B**) Morphometric gating on intact leukocyte populations (*FSC-A* vs. *SSC-A*). (**C–D**) Determination of specific myeloid cell populations through marker analysis (antibodies listed in ESM Table 1): (**C**) Dendritic cells and myeloid subpopulations defined by CD11c and CD11b expression, and (**D**) Macrophage (F4/80+) and granulocyte/neutrophil (GR1+) populations.

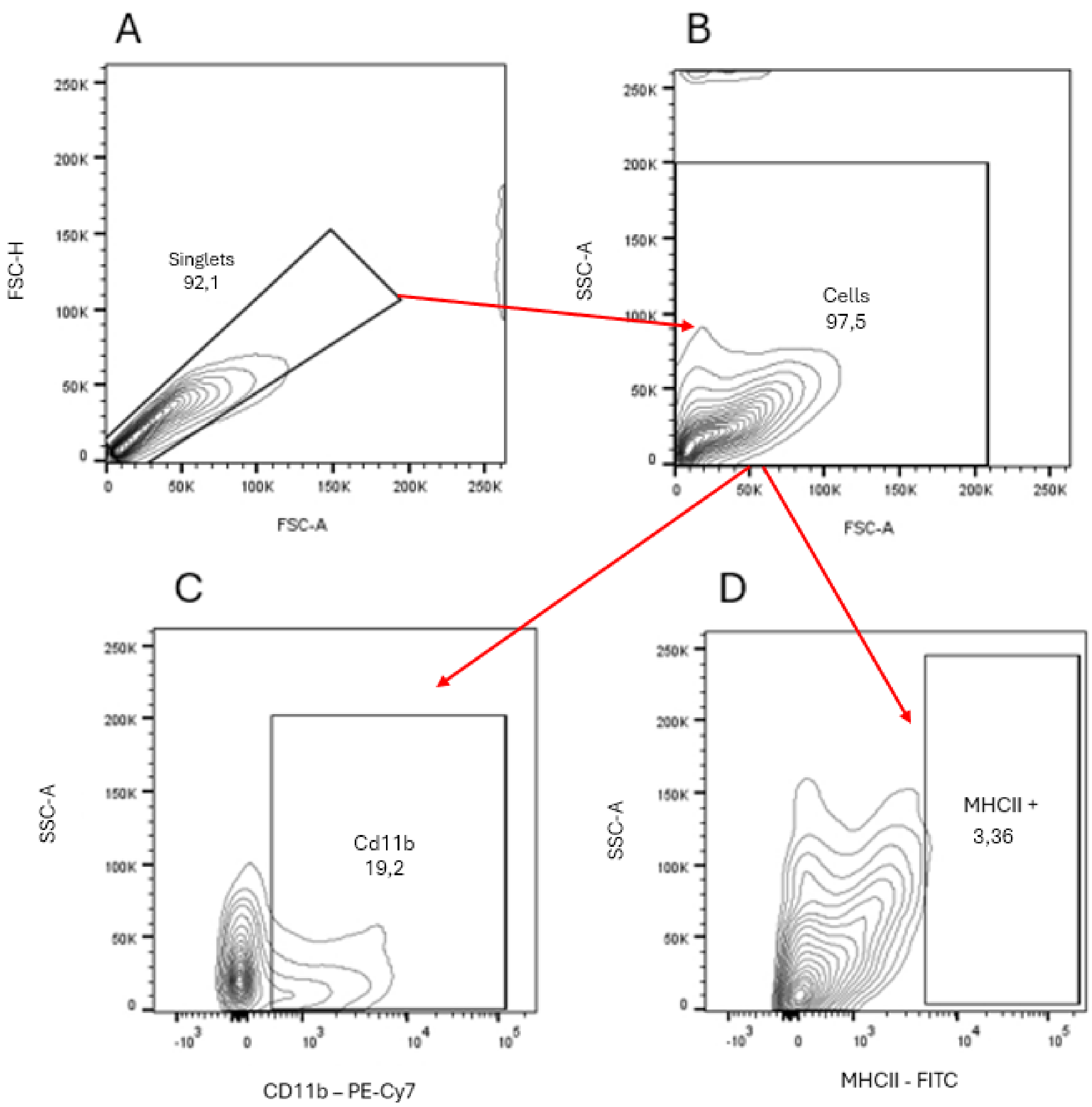

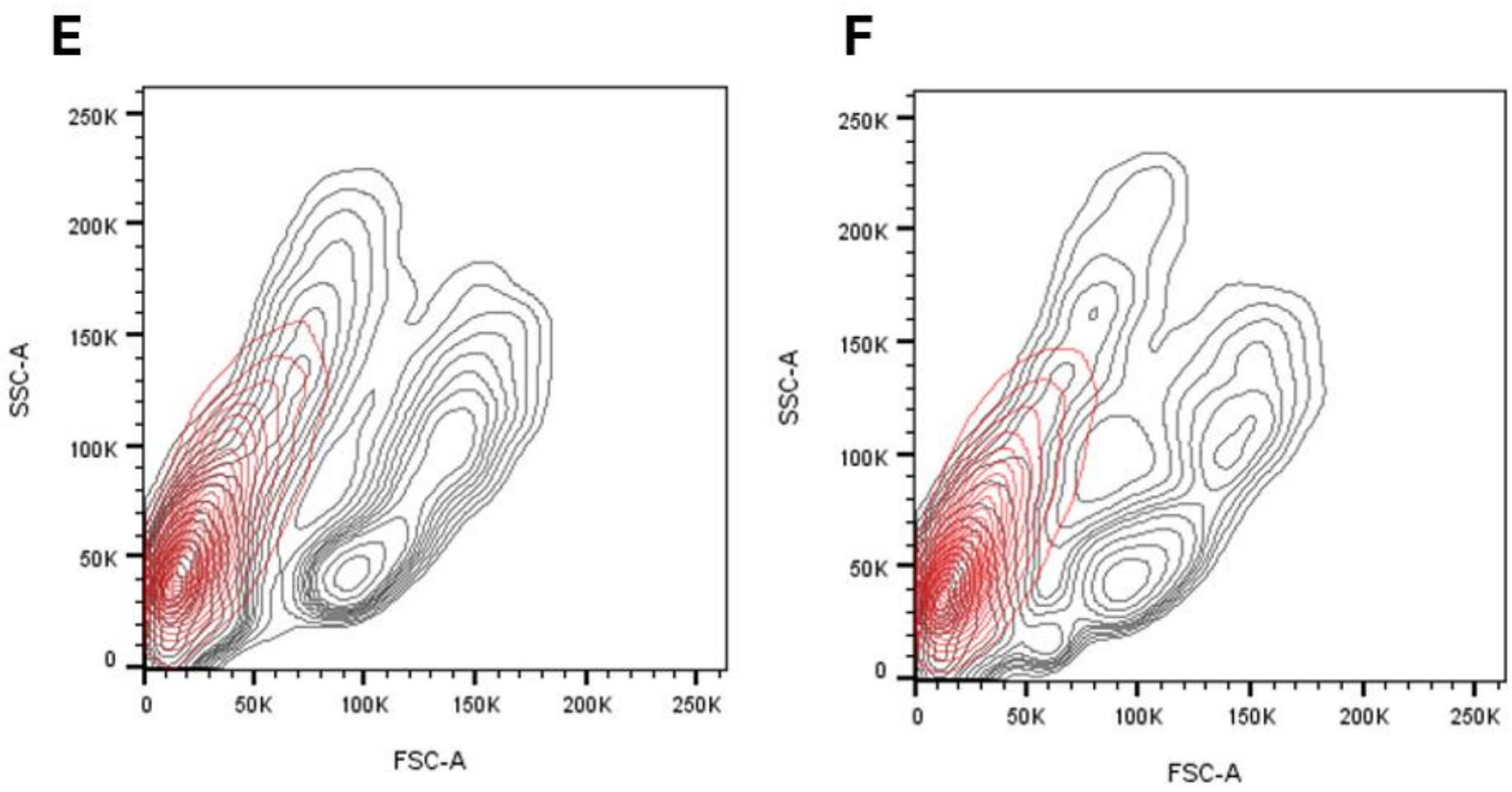


**Gating strategy in <u>Figure 9g-h</u>**. The image is representative of one of the phenotyping analyses performed in this study. Red arrows indicate the sequential gating hierarchy. (**A**) Exclusion of doublets. (**B**) Selection of the cell profile to be analyzed. (**C**) Determination of the population to be analyzed by marker analysis. (**D**) Determination of the amount of CFSE-labeled fungus phagocytosed. (**E–F**) *In vitro* quantification of BMDM phagocytosis rates when challenged with *Sporothrix brasiliensis* at a multiplicity of infection (MOI) of 1:3: evaluations were recorded at the 2-hour (**E**) and 4-hour (**F**) time points.

**ESM Fig. 4:** Photos of BHI agar and PCR gel confirming the *Sporothrix brasiliensis* strain present in a culture of bone marrow-derived macrophages:

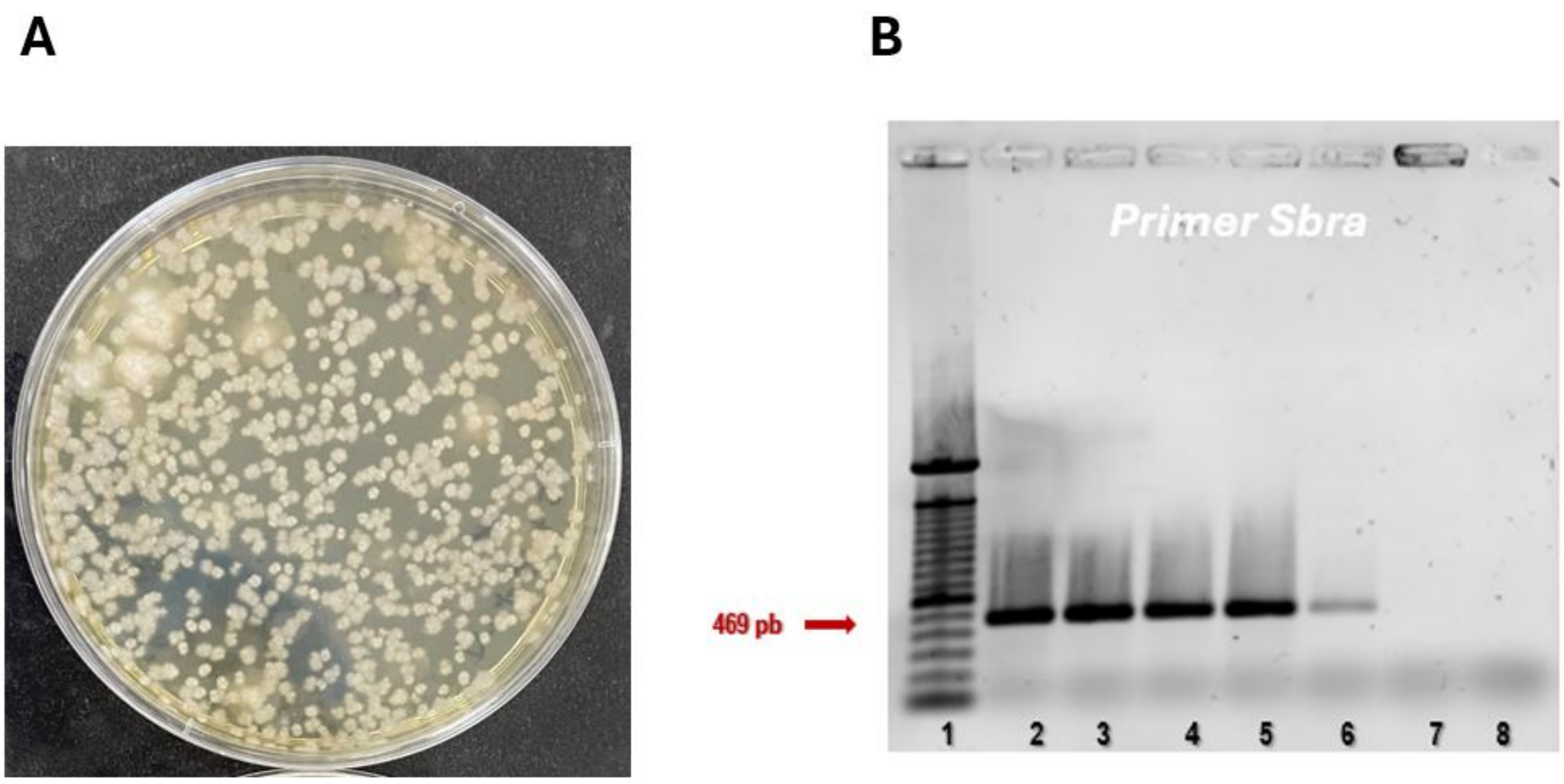


**Confirmation of bone marrow colonization by *Sporothrix brasiliensis*.** (**A**) Representative Brain Heart Infusion (BHI) agar plate showing fungal colony growth from bone marrow cell culture supernatants isolated from infected mice. (**B**) Molecular identification of fungal isolates by PCR targeting the calmodulin (*CAL*) gene (469 bp product), analyzed by electrophoretic separation on a 1% agarose gel for 120 min. Lane 1: 100 bp DNA ladder (molecular weight marker); Lane 2: Positive control (*Sporothrix brasiliensis* reference strain M1168); Lanes 3–6: Representative fungal lysate samples isolated from experimental groups (Lane 3: CT + Sb; Lane 4: CT + Sb + R; Lane 5: DT + Sb; Lane 6: DT + Sb + R); Lane 7: Specificity control (*Candida albicans*); Lane 8: Negative control (nuclease-free water). Red arrow indicates the expected 469 bp specific amplicons.

**Reference for the Species-specific primer**: Rodrigues AM, De Hoog GS, De Camargo ZP. (2015) Molecular Diagnosis of Pathogenic *Sporothrix* Species. PLOS Neglected Tropical Diseases. December 1, 2015. https://doi.org/10.1371/journal.pntd.0004190

**ESM Fig. 5: Tissue and systemic cytokine profiles determined by Cytometric Bead Array (CBA) during *Sporothrix brasiliensis* infection (from Fig. 6).**

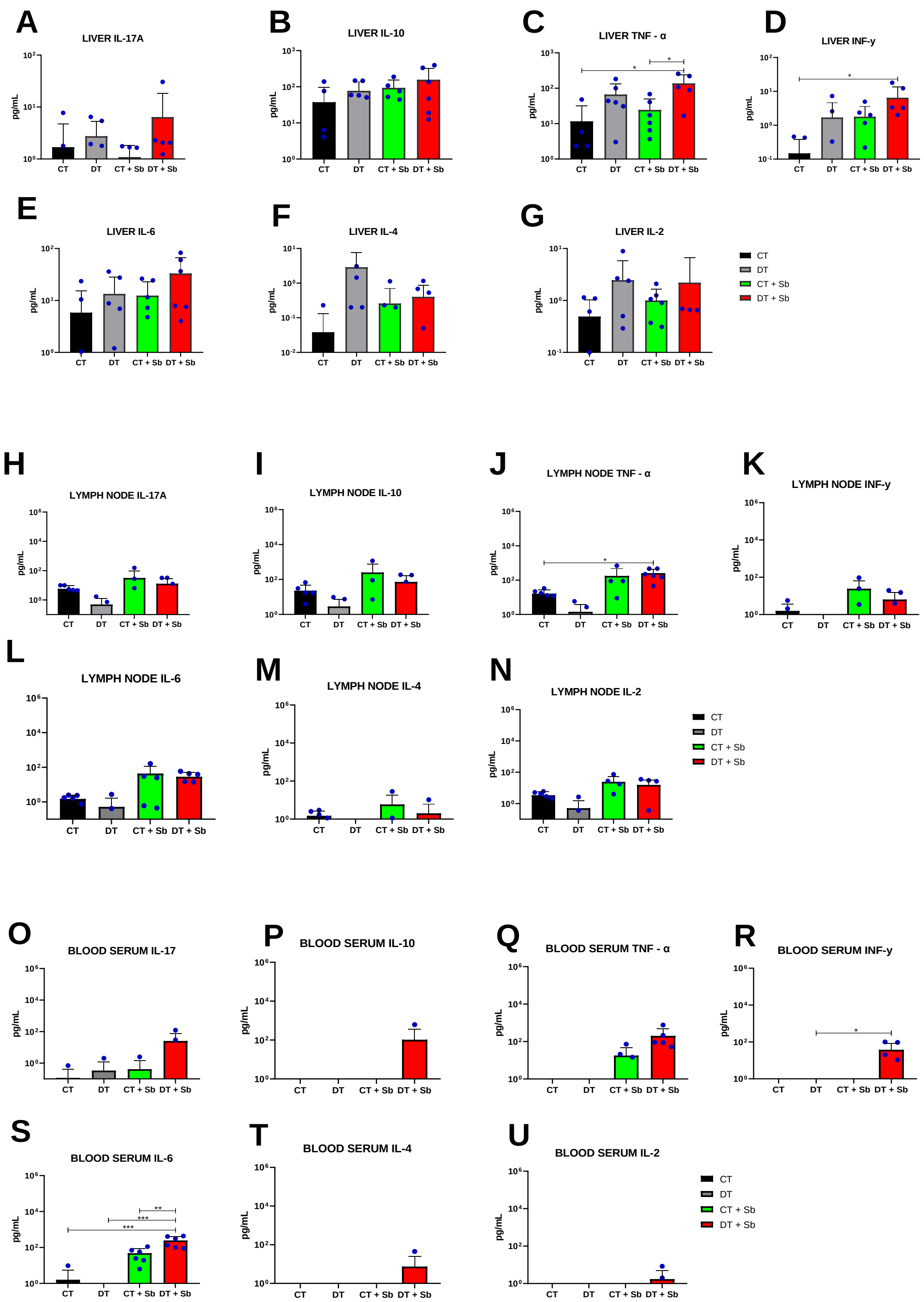


**Tissue and systemic cytokine profiles determined by Cytometric Bead Array (CBA) during *Sporothrix brasiliensis* infection.** Quantitative protein levels (pg/mL) were

evaluated 14 days post-infection in healthy and diabetic mice across three distinct compartments: hepatic tissue (**A–G**), draining lymph nodes (**H–N**), and blood serum (**O–U**). Cytokines analyzed per compartment include: (**A, H, O**) IL-17A; (**B, I, P**) IL-10; (**C, J, Q**) TNF-α; (**D, K, R**) IFN-γ; (**E, L, S**) IL-6; (**F, M, T**) IL-4; and (**G, N, U**) IL-2. Experimental groups are defined as: uninfected control (CT, black bars), uninfected diabetic (DT, gray bars), normoglycemic infected (CT+Sb, green bars), and diabetic infected (DT+Sb, red bars). Data are expressed as mean ± SEM. Statistical significance was determined by Ordinary one-way ANOVA followed by Tukey's multiple comparisons test; n = 6 animals per group. Asterisks indicate statistically significant differences between the linked groups ($p < 0.05$). Note that major significant modifications are restricted to the pro-inflammatory TNF-α, IFN-γ, and IL-6 axis, while no significant modulations were observed for IL-17A, IL-10, IL-4, and IL-2 in most evaluated sites.
